# Comprehensive pan-cancer genomic landscape of *KRAS* altered cancers and real-world outcomes in solid tumors

**DOI:** 10.1038/s41698-022-00334-z

**Published:** 2022-12-09

**Authors:** Jessica K. Lee, Smruthy Sivakumar, Alexa B. Schrock, Russell Madison, David Fabrizio, Ole Gjoerup, Jeffrey S. Ross, Garrett M. Frampton, Pavel Napalkov, Meagan Montesion, Jennifer L. Schutzman, Xin Ye, Priti S. Hegde, Misako Nagasaka, Geoffrey R. Oxnard, Ethan S. Sokol, Sai-Hong Ignatius Ou, Zhen Shi

**Affiliations:** 1 Foundation Medicine Inc., Cambridge, MA USA; 2 Upstate Medical University, Syracuse, NY USA; 3 Genentech, Inc., South San Francisco, CA USA; 4 Chao Family Comprehensive Cancer Center, University of California Irvine School of Medicine, Orange, CA USA

**Keywords:** Molecular medicine, Predictive markers, Cancer genomics, Outcomes research

## Abstract

Recent clinical development of KRAS inhibitors has heightened interest in the genomic landscape of *KRAS*-altered cancers. We performed a pan-cancer analysis of *KRAS*-altered samples from 426,706 adult patients with solid or hematologic malignancies using comprehensive genomic profiling; additional analyses included 62,369 liquid biopsy and 7241 pediatric samples. 23% of adult pan-cancer samples had *KRAS* alterations; 88% were mutations, most commonly *G12D/G12V/G12C/G13D/G12R*, and prevalence was similar in liquid biopsies. Co-alteration landscapes were largely similar across *KRAS* mutations but distinct from *KRAS* wild-type, though differences were observed in some tumor types for tumor mutational burden, PD-L1 expression, microsatellite instability, and other mutational signatures. Prognosis of *KRAS-*mutant versus other genomic cohorts of lung, pancreatic, and colorectal cancer were assessed using a real-world clinicogenomic database. As specific KRAS inhibitors and combination therapeutic strategies are being developed, genomic profiling to understand co-alterations and other biomarkers that may modulate response to targeted or immunotherapies will be imperative.

## Introduction

The Kirsten rat sarcoma viral oncogene homolog (*KRAS*) gene belongs to the rat sarcoma (RAS) family of oncogenes that also includes Harvey rat sarcoma (*HRAS*) and neuroblastoma rat sarcoma (*NRAS*) viral oncogene homologs and, when mutated, can initiate or promote cancer growth^1–5^. Activating mutations in *KRAS* are among the most prevalent oncogenic driver mutations in human cancers and are associated with tumorigenesis as well as aggressive tumor growth. Despite decades of research, KRAS had been an “undruggable” target until the landmark discovery of covalent inhibitors specific for KRAS G12C^6^. Clinical trials of mutant-specific KRAS G12C inhibitors have shown promising activity^7–12^. Sotorasib and adagrasib each received FDA breakthrough designation for the treatment of advanced or metastatic non-small cell lung cancer (NSCLC) harboring a *KRAS* G12C mutation, and sotorasib has now received marketing authorization in the US and other countries for the treatment of certain patients with *KRAS* G12C NSCLC^13^. Besides the clinical development of direct covalent KRAS G12C inhibitors, there are significant efforts underway to develop other mutant-specific and pan-KRAS inhibitors, and inhibitors that target upstream of the RAS pathway (SOS1, SHP2)^14–16^.

Here we performed a comprehensive pan-cancer genomic analysis to identify the incidence of *KRAS* alterations across 24 tumor types, the distribution of *KRAS* alterations inclusive of and beyond *G12C*. We evaluated the genomic co-alteration landscapes and immune biomarker patterns in association with different *KRAS* mutations in terms of tumor mutational burden (TMB), PD-L1 expression, co-alterations, and mutational signatures that may modulate response to KRAS inhibitors, immune checkpoint inhibitors (ICI) or other therapies. We also interrogated a real-world clinicogenomic database (CGDB) to assess prognostic implications for *KRAS* mutated subsets compared to other genomically defined cohorts of NSCLC, colorectal cancer (CRC), and pancreatic ductal adenocarcinoma (PDAC).

## Results

### Prevalence of *KRAS* alterations in adult and pediatric cancers

**Fig. 1:**
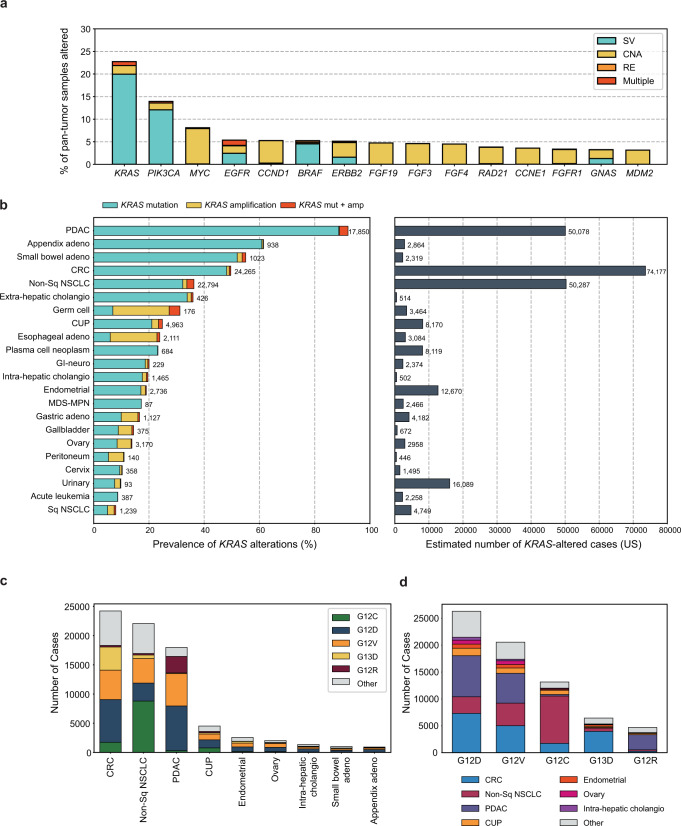
Prevalence of *KRAS* alterations among adult patients with cancer. **a** In the Foundation Medicine (FM) dataset of tissue or hematologic samples from 426,706 adult patients with cancer, *KRAS* was the most frequently altered oncogene with alterations in 23% of samples. A longtail of other top frequently altered oncogenes is shown; bar colors indicate alteration classes, SV: short variant mutation (e.g., substitutions, indels), CNA: copy number alteration, RE: rearrangement. **b** Prevalence in the FM dataset (left) and incidence estimates in the United States (right) of *KRAS* alterations in common adult tumor types (Supplemental Table 1). *KRAS* alterations are most prevalent in PDAC, appendix adenocarcinoma, small bowel and CRC tumor types. The highest incidence of *KRAS* alterations is estimated in CRC, non-Sq NSCLC and PDAC. **c***,* **d** Number of cases in the FM database with *KRAS* mutations among (**c**) the 8 top indications with highest incidence of *KRAS* alterations and carcinoma of unknown primary (CUP) and **d** the 5 most common *KRAS* mutant isoforms.

A total of 426,706 unique tissue or hematologic samples from adult patients with cancer were submitted for testing during routine clinical care from December 2013 to December 2021. *KRAS* was the most frequently altered oncogene with alterations identified in 97,062 (23%) pan-tumor tissue samples. The vast majority (88%) of the alterations were mutations (99.7% substitutions and 0.2% insertion or deletions [indel]), 8.4% were *KRAS* amplifications and 3.8% a combination of mutation with amplification (Fig. 1a). The estimated incidence for *KRAS* altered cancers in the US based on this prevalence data is highest in CRC with almost 75,000 cases followed by PDAC and non-squamous (non-Sq) NSCLC (>50,000 cases each; Fig. 1b and Supplemental Table 1). *KRAS* G12D (29%), G12V (23%), G12C (15%), G13D (7%), and G12R (5%) were the five most common *KRAS* mutant isoforms together accounting for ~80% of all *KRAS* alterations. The tumor types with the highest prevalence of *KRAS* mutations (*KRAS*m) were PDAC (92%), appendiceal adenocarcinoma (61%), small bowel adenocarcinoma (SBA, 53%), CRC (49%), and non-squamous (non-Sq) NSCLC (35%) (Supplemental Table 2); CRC, non-Sq NSCLC, PDAC together represented 71% (63,480/88,907) of the *KRAS*m pan-tumor population (Fig. 1c, d). Additionally, several other tumor types harbored *KRAS*m including most notably: extrahepatic cholangiocarcinoma (35%), carcinoma of unknown primary (CUP, 22%), intrahepatic cholangiocarcinoma (ICC, 18%), endometrial (17%), gastric (11%), as well as breast (2.1%) and prostate (1.3%) carcinomas (Supplemental Table 2). *KRAS* amplification (median 11 copies, range 5-421) was rare across most tumor types but common in germ cell tumors (24%) and esophageal adenocarcinoma (18%).

**Fig. 2:**
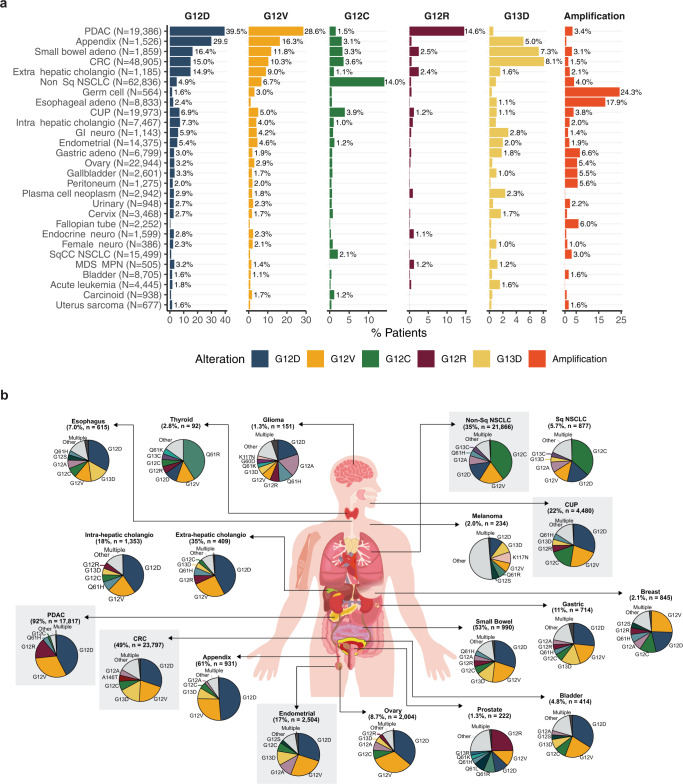
Prevalence of *KRAS* alteration subtypes by tumor type. **a** Bar graph showing the prevalence of the most common types of *KRAS* alterations across indications. Disease subtypes are ordered based on the cumulative prevalence across the six highlighted *KRAS* alteration subtypes. Only tumor types with at least 250 total samples and a total *KRAS* alteration prevalence of at least 5% are shown. **b** Anatomic visualization of the prevalence of *KRAS* mutant isoforms. Percentage of total cases with *KRAS* mutations is shown for each tumor type and the corresponding ‘n’ for number of *KRAS* mutated cases. The four major tumor types assessed in this study as well as carcinoma of unknown primary (CUP) are highlighted in gray and represent the largest *KRAS* mutant populations. Major tumor types with relatively low prevalence of *KRAS* alterations such as breast, prostate, glioma, and melanoma are also shown. The body part background was custom designed by www.slideteam.net [slideteam.net].

Although the *KRAS*m distribution varied across adult tumor types, similar patterns of *KRAS*m were observed among tumors originating from similar tissue types*. KRAS* G12C was the most common variant among NSCLC (40% and 36% of *KRAS*m non-Sq and Sq, respectively). Gastrointestinal cancers (including CRC and cancers of the esophagus, stomach, small bowel, and appendix) also share a similar profile where *KRAS* G12D and G12V were the top two common variants. *KRAS* G12D was also most common among many other tumor types including PDAC (43%), and endometrial (30%), and *KRAS* G12V was the second most common in the majority of tumor types studied and the most common *KRAS*m in breast (26%) (Supplemental Table 2 and Fig. 2a, b). *KRAS*m isoforms were all largely clonal indicating that these mutations are likely truncal in all four major tumor types studied (Supplemental Fig. 1 and Supplemental Table 3). For comparative analysis, 62,369 samples underwent liquid genotyping from May 2016 to December 2021. *KRAS* alterations were detected in 15% of all liquid biopsies, and in 19% of liquid biopsies with elevated tumor fraction, including mutations (95.5%), amplification (3.2%) and a combination of mutation with amplification (1.3%; Supplemental Fig. 2A). *KRAS* G12D (26%), G12V (20%), G12C (17%), G13D (6%), and G12A (4%) were the five most common *KRAS*m detected by liquid genotyping, which was similar to the pan-tumor distribution seen in tissue (Supplemental Fig. 2B).

Available clinicogenomic characteristics of the four major *KRAS*m tumor types (NSCLC, CRC, PDAC, endometrial) selected based on prevalence, incidence, and the landscape of KRAS inhibitor development, stratified by major *KRAS* isoforms, are shown in Table 1, and additional tumor types as well as pan-cancer analysis are described in Supplemental Table 4. The pan-tumor *KRAS* G12C subset was notably distinct from cases with non-G12C mutations or *KRAS* WT, with patients carrying G12C mutant tumors being older (median age 66 vs 64 vs 64 years), more often female (58% vs 52% vs 54%), having higher TMB (32% vs 12% vs 16% with TMB ≥ 10 mutations/Mb), and a greater fraction with high PD-L1 expression (37% vs 14% vs 14%) (all comparisons *p* < 0.001).

**Table 1:** Clinico-pathologic characteristics of patients with four major *KRAS* mutated tumors by *KRAS* mutation subtype.

CRC (*N* = 23,797 *KRAS* mutated cases)								
	*KRAS* G12C	*KRAS* G12D	*KRAS* G12V	*KRAS* G13D	*KRAS* WT	*KRAS* non-G12C	G12C vs non-G12C	G12C vs. WT
# cases	1739	7312	5026	3963	24,640	22,058	–	–
Median age (range)	60 (22–89+)	60 (18–89+)	61 (20–89+)	61 (19–89+)	60 (18–89+)	60 (18–89+)	0.21	0.27
≥65 years old	35% (608)	38% (2761)	38% (1902)	38% (1518)	37% (9194)	38% (8349)	0.04	0.10
Sex M:F %	52:48	53:47	53:47	53:47	57:43	53:47	0.78	*P* < 0.001
Median TMB (range)	3.8 (0–90)	3.8 (0–219)	3.5 (0–373)	3.8 (0–608)	3.8 (0–865)	3.8 (0–608)	0.59	0.07
TMB ≥ 10 mutations/Mb (*n*)	4.7% (81)	6.6% (482)	3.2% (163)	9.5% (377)	11% (2670)	6.8% (1510)	0.002	*P* < 0.001
MSI-H % (*n*)	0.65% (11/1691)	3.4% (237/7048)	0.57% (28/4870)	5.6% (217/3860)	7.5% (1784/23,929)	3.2% (690/21,347)	*P* < 0.001	*P* < 0.001
PD-L1 1–49%	15% (68/445)	17% (315/1832)	13% (173/1282)	16% (166/1022)	13% (836/6552)	16% (880/5595)	0.92	0.21
PD-L1 ≥ 50%	1.6% (7/445)	1.4% (26/1832)	0.47% (6/1282)	1.2% (12/1022)	2.0% (134/6552)	1.0% (58/5595)	0.50	0.72

Non-Sq NSCLC (*N* = 21,866 *KRAS* mutated cases)								
	*KRAS* G12C	*KRAS* G12D	*KRAS* G12V	*KRAS* G12A	*KRAS* WT	*KRAS* non-G12C	G12C vs non-G12C	G12C vs. WT
# cases	8790	3085	4197	1548	40,042	13,076	–	–
Median age (range)	67 (24–89+)	69 (23–89+)	68 (24–89+)	69 (30–89+)	67 (18–89+)	68 (23–89+)	*P* < 0.001	*P* < 0.001
≥65 years old	60% (5282)	64% (1986)	62% (2584)	65% (1000)	59% (23,651)	62% (8130)	0.003	0.08
Sex M:F %	40:60	44:56	42:58	42:58	49:51	43:57	*P* < 0.001	*P* < 0.001
Median TMB (range)	7.8 (0–104)	5.0 (0–164)	6.3 (0–937)	6.3 (0–68)	5.2 (0–1765)	6.3 (0–1255)	*P* < 0.001	*P* < 0.001
TMB ≥ 10 mutations/Mb (*n*)	40% (3546)	24% (754)	34% (1412)	31% (485)	32% (12,842)	33% (4260)	*P* < 0.001	*P* < 0.001
MSI-H % (*n*)	0.20% (17/8473)	0.30% (9/2974)	0.20% (8/4054)	0.13% (2/1491)	0.35% (136/38,548)	0.26% (33/12,605)	0.59	0.03
PD-L1 1–49%	28% (1067/3879)	29% (377/1285)	28% (506/1820)	29% (189/655)	30% (4937/16,351)	28% (1578/5633)	0.72	0.001
PD-L1 ≥ 50%	44% (1690/3879)	37% (477/1285)	39% (705/1820)	37% (244/655)	29% (4769/16,351)	38% (2147/5633)	*P* < 0.001	*P* < 0.001

PDAC (*N* = 17,817 *KRAS* mutated cases)								
	*KRAS* G12C	*KRAS* G12D	*KRAS* G12V	*KRAS* G12R	*KRAS* WT	*KRAS* non-G12C	G12C vs non-G12C	G12C vs. WT
# cases	300	7654	5542	2825	1536	17,517	–	–
Median age (range)	67 (37–87)	66 (21–89+)	66 (28–89+)	66 (29–89+)	64 (23–89+)	66 (21–89+)	0.42	*P* < 0.001
≥65 years old	59% (176)	56% (4253)	55% (3065)	59% (1668)	49% (757)	56% (9888)	0.64	0.02
Sex M:F %	59:41	53:47	52:48	49:51	59:41	52:48	0.06	0.95
Median TMB (range)	1.7 (0–21)	1.3 (0–344)	1.3 (0–166)	1.3 (0–211)	1.7 (0–6906)	1.3 (0–355)	0.001	0.55
TMB ≥ 10 mutations/Mb (*n*)	2.0% (6)	1.0% (78)	0.79% (44)	0.85% (24)	5.5% (85)	1.0% (177)	0.32	0.03
MSI-H % (*n*)	0.34% (1/292)	0.37% (28/7472)	0.20% (11/5387)	0.15% (4/2759)	1.5% (23/1487)	0.32% (55/17,065)	0.74	0.37
PD-L1 1%-49%	35% (29/84)	33% (706/2118)	30% (457/1528)	25% (187/755)	24% (98/410)	31% (1484/4821)	0.64	0.16
PD-L1 ≥50%	2.4% (2/84)	7.7% (164/2118)	4.6% (71/1528)	6.0% (45/755)	6.6% (27/410)	6.3% (302/4821)	0.35	0.37

Endometrial (*N* = 2504 *KRAS* mutated cases)								
	*KRAS* G12C	*KRAS* G12D	*KRAS* G12V	*KRAS* G12A	*KRAS* WT	*KRAS* non-G12C	G12C vs non-G12C	G12C vs. WT
# cases	175	771	657	291	11,639	2329	–	–
Median age (range)	64 (35–89+)	63 (27–89+)	63 (24–89+)	64 (33–89+)	66 (23–89+)	64 (24–89+)	0.95	0.01
≥65 years old	46% (81)	44% (340)	44% (286)	49% (144)	56% (6484)	46% (1082)	1.0	0.03
Sex M:F %	0.57:99	0:100	0:100	0:100	0:100	0:100	0.21	0.03
Median TMB (range)	3.8 (0–54)	3.8 (0–509)	2.6 (0–384)	3.8 (0–393)	2.6 (0–908)	3.8 (0–733)	0.95	0.01
TMB ≥ 10 mutations/Mb (*n*)	30% (52)	34% (264)	21% (141)	31% (89)	16% (1919)	33% (759)	0.78	*P* < 0.001
MSI-H % (*n*)	26% (43/166)	31% (225/718)	19% (120/621)	27% (72/270)	13% (1459)	29% (619)	0.80	*P* < 0.001
PD-L1 1–49%	39% (25/64)	26% (66/258)	24% (54/224)	38% (39/104)	28% (1135/4005)	30% (245/814)	0.32	0.09
PD-L1 ≥50%	4.7% (3/64)	1.2% (3/258)	1.3% (3/224)	2.9% (3/104)	1.8% (73/4005)	2.0% (16/814)	0.32	0.14

Ordinal relationships were examined using the Mann–Whitney *U* test; categorical relationships were examined using Fisher’s exact with correction for multiple comparisons. PD-L1 expression was only available for a subset of cases.

*KRAS* WT includes samples WT for *KRAS* mutations and amplifications.

Analysis of a pediatric cohort was also performed. Among tissue or hematologic samples from pediatric patients (*n* = 7241 unique patients), *KRAS* alterations were present in 5.5% of samples. The pediatric group with the largest number of *KRAS*m cases was acute leukemia where diverse *KRAS*m were represented with *KRAS* G13D and G12D being the most common. *KRAS*m were most prevalent in CRC (28%), germ cell tumors (20%), and myelodysplastic-myeloproliferative neoplasms (MDS-MPN, 20%); however, the total number of patients with *KRAS*m are small due to their rarity (Supplemental Fig. 3). Based on the estimated incidence rates of different pediatric tumors in the US and prevalence of *KRAS* alterations observed in our cohort, we estimate the highest incidence of *KRAS* altered cases to be in acute leukemia (*n* = 553), followed by colorectal (*n* = 88), ovary (*n* = 71), and glioma (*n* = 64; Supplemental Table 1).

### Co-alteration landscapes, mutational signatures, and immunotherapy biomarkers

**Fig. 3:**
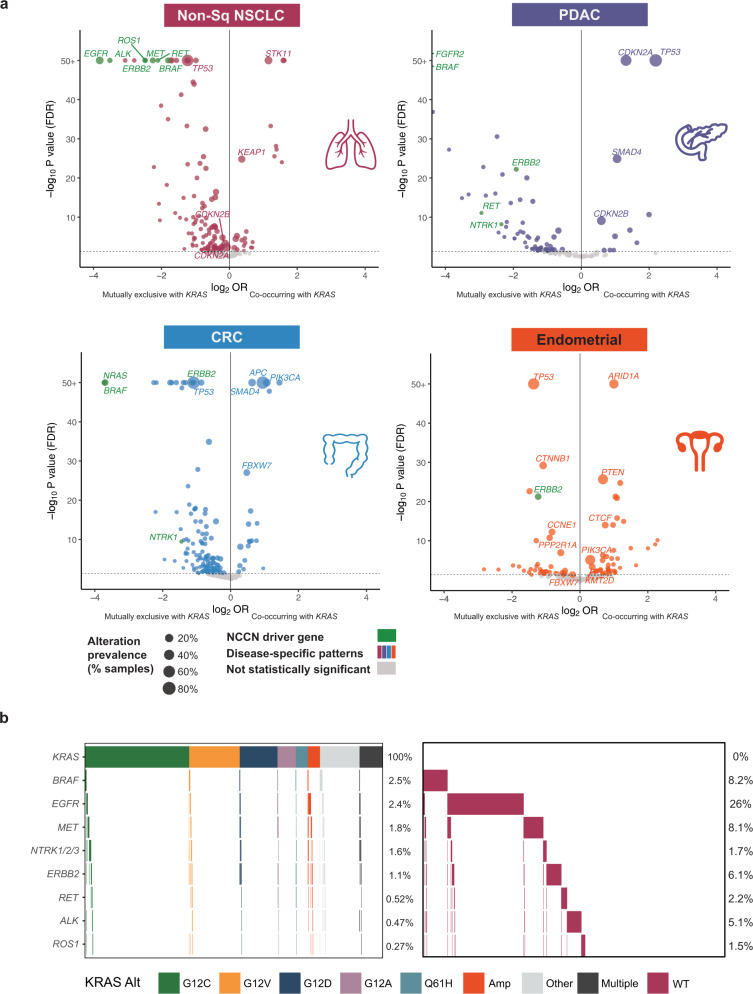
Co-occurrence of gene alterations among *KRAS* altered non-Sq NSCLC, PDAC, CRC and endometrial cancer. **a** The prevalence of alterations was compared for *KRAS* altered and *KRAS* wild type (WT) non-Sq NSCLC (*N* = 62,836), PDAC (*N* = 19,386), CRC (*N* = 48,905), and endometrial (*N* = 14,375) tumor samples. For each tumor cohort, only genes altered in at least 50 cases and targeted across all the assay versions were included. For each gene, substitutions, short insertions/deletions, rearrangements, and copy number changes of known or likely functional significance detected using our assay were included. Driver genes highlighted in the National Comprehensive Cancer Network (NCCN) Guidelines as well as genes altered at a high prevalence (≥10%) are labeled for each volcano plot. Alterations in known driver oncogenes (labeled in green) tend to be mutually exclusive with *KRAS* alterations (left side of plots) in all four major tumor types studied (*p* ≤ 0.05; Odd’s ratio <1). **b** Oncoprints showing the frequency and mutual exclusivity of NCCN driver genes in *KRAS* altered (*N* = 22,794) vs *KRAS* WT (*N* = 40,042) non-Sq NSCLC. Fisher’s exact test was applied to assess patterns of co-occurrence and mutual exclusivity between *KRAS* and other genes alterations. *P* values were corrected with the Benjamini–Hochberg FDR method.

Among four major *KRAS* altered tumor types, volcano plots of co-occurring or mutually exclusive alterations with *KRAS* are shown in Fig. 3a (see also Supplemental Tables 5–8). In non-Sq NSCLC, CRC and endometrial cancer *TP53* was the most frequently altered gene and tended to be mutually exclusive from *KRAS*, whereas *TP53* alterations tended to co-occur with *KRAS* in PDAC. Consistent with *KRAS* being a key oncogenic driver, alterations in *KRAS* were highly mutually exclusive with other driver alterations in the RTK/MAPK pathway, including *EGFR*, *ALK*, *MET*, *ERBB2*, *BRAF*, *RET*, and *ROS1* in non-Sq NSCLC (Fig. 3b); *BRAF*, *FGFR2, ERBB2, RET* and *NTRK1* in PDAC; *NRAS*, *BRAF*, and *ERBB2* in CRC; and *ERBB2* in endometrial. Genes with co-alterations notably enriched in *KRAS*m subsets included *STK11* in non-Sq NSCLC, *PIK3CA* and *APC* in CRC, and *ARID1A, PTEN*, and *PIK3CA* in endometrial tumors. Of note, largely similar patterns of co-mutations and mutual exclusivity were observed when limiting to a subset of microsatellite stable (MSS) CRC and endometrial tumors (Supplemental Fig. 4 and Supplemental Tables 6 and 8). Volcano plots for four additional tumor types (Sq NSCLC, SBA, ICC, and appendix adenocarcinoma) also support mutual exclusivity of *KRAS* with other driver alterations (Supplemental Fig. 5 and Supplemental Tables 9–12).

For the top differentially occurring genes with *KRAS*, the co-alteration landscape was largely similar across *KRAS*m isoforms, but distinct for *KRAS* WT within a given tumor type (Supplemental Fig. 6). Statistical analysis of the three most common *KRAS*m isoforms revealed additional allele-specific differences, especially in relatively rare gene alterations, within specific tumor types (Supplemental Table 13). For example, alterations in *GNAS* were more common in *KRAS* G12D compared to *KRAS* G12C mutated non-Sq NSCLC (5.7% vs. 2.0% respectively; FDR *p* < 10^−4^). Co-mutations in other MAPK/PI3K pathway genes tended to be more frequent in *KRAS* G13D compared to *KRAS* G12D mutated CRCs (*NF1*: 3.0% vs. 1.2%, *AKT1*: 2.0% vs. 0.8%, *BRAF*: 1.7% vs. 0.7% respectively; all FDR *p* < 10^−4^). Similarly, in endometrial tumors, *KRAS* G13D mutated tumors showed a higher prevalence of *NF1* and *PTEN* alterations compared to *KRAS* G12D (*NF1*: 14.0% vs. 6.1%, FDR *p* = 0.005; *PTEN*: 70.6% vs. 55.1%, *p* = 0.0006, respectively). In PDAC, *ARID1A* and *ERBB2* alterations were detected more commonly in *KRAS* G12D mutated tumors compared to *KRAS* G12R mutated tumors (*ARID1A*: 9.7% vs. 4.6%, FDR *p* < 10^−4^; *ERBB2*: 2.2% vs. 1.0%, FDR *p* = 0.0003).

**Fig. 4:**
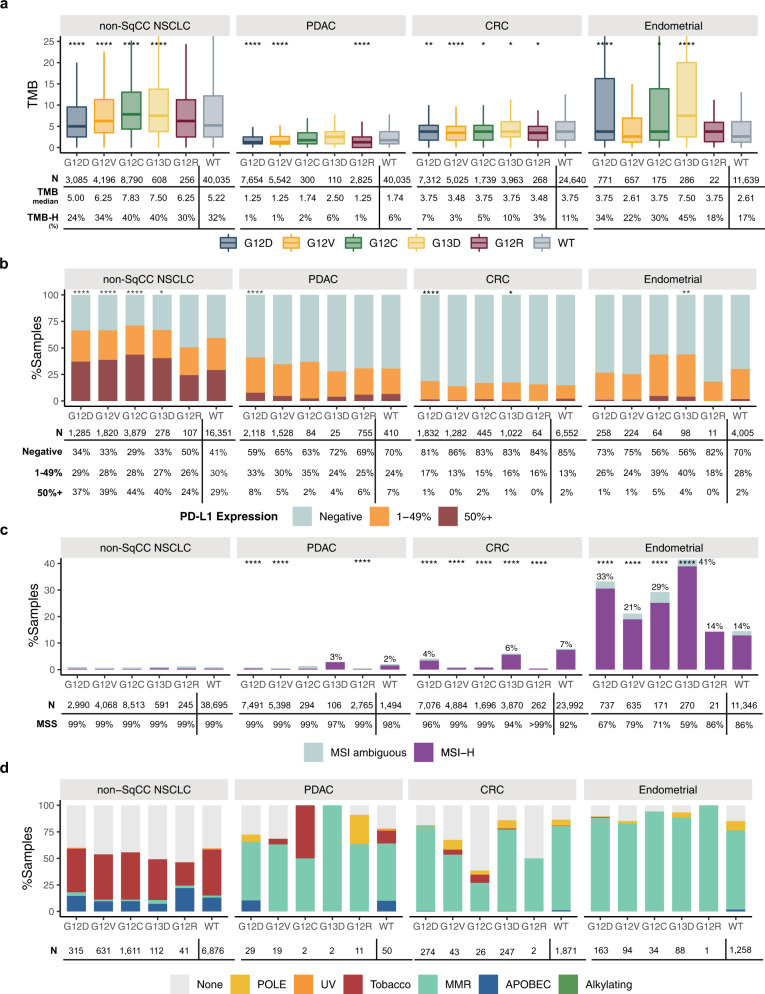
Immunotherapy biomarkers and mutational signatures associated with *KRAS*m isoforms in non-Sq NSCLC, PDAC, CRC, and endometrial cancers. **a** Box plots showing the distribution of tumor mutational burden (TMB) in *KRAS*m and *KRAS* WT tumors. TMB is higher in *KRAS*m vs *KRAS* wild-type (WT) non-Sq NSCLC and endometrial cancer, and in particular for G12C and G12D subsets of non-Sq NSCLC and G13D in endometrial cancer. Each box plot displays the interquartile range (IQR), with the lower boundary representing the 25th percentile and the upper boundary representing 75th percentile. The line within the box displays the median and the whiskers extend to ±1.5 x IQR. **b** PD-L1 expression was relatively consistent across *KRAS*m and WT subsets for the four major tumor types. In non-Sq NSCLC PD-L1 high expression was enriched in G12D/V/C and G13D subsets relative to WT and in endometrial tumors, any PD-L1 expression was enriched in G12C and G13D relative to WT. **c** Microsatellite instability (MSI) was low across non-Sq NSCLC and PDAC. In CRC, MSI-high was enriched in *KRAS* WT compared to *KRAS*m subsets, whereas in endometrial tumors, G12D/V/C and G13D subsets had elevated MSI-high compared to WT. Each *KRAS* mutation isoform was compared against WT with *p* value thresholds: 0.0001: ****, 0.001: ***, 0.01: **, 0.05: *. **d** Six mutational signatures were assessed for *KRAS*m isoforms. Tobacco signature was common across *KRAS*m and WT non-Sq NSCLC and mismatch repair (MMR) was common across PDAC, CRC and endometrial tumors. Only a subset of cases were able to be assessed for mutational signatures and number of cases is shown below each bar.

In non-Sq NSCLC, *KRAS* G12C mutated tumors were enriched for high TMB ≥ 10 mutations/Mb (40% vs 33% vs 32% for *KRAS* non-G12C and WT, both *p* < 0.001) and for high PD-L1 expression (44% vs 38% for *KRAS* non-G12C and 29% for WT, both *p* < 0.001), whereas *KRAS* G12D mutated tumors had lower incidence of elevated TMB (24% with TMB ≥ 10 mutations/Mb) relative to other *KRAS*m isoforms assessed (Fig. 4a, b and Table 1). In Sq NSCLC the same trends were observed, but most differences were not statistically significant (Supplemental Table 4). Relative to non-Sq NSCLC, the fraction of samples positive for high PD-L1 expression in *KRAS*m CRC, PDAC, and endometrial was low (Fig. 4b), and relatively few ICC, SBA and appendiceal samples were tested for PD-L1 (Supplemental Table 4). High TMB was also less frequent in *KRAS*m CRC, PDAC, ICC, SBA, and appendiceal compared to NSCLC; however, endometrial was similar to NSCLC with 21–34% of samples having TMB ≥ 10. In endometrial cancers, high TMB was enriched in *KRAS* G13D, G12D, G12C, and G12A (45%, 34%, 30%, and 31%) compared to G12V and WT (21% and 16%, *p* < 0.05 for all comparisons; Table 1 and Fig. 4a). High MSI was rare (<1%) across non-Sq NSCLC subtypes, and infrequent in PDAC and CRC relative to endometrial cancer where 14–39% of *KRAS*m isoforms were MSI high. Across tumor types, *KRAS* G13D and G12D were associated with the highest levels of MSI high relative to other *KRAS*m isoforms (Fig. 4c). We also investigated *KRAS* allele-specific patterns of the loss of heterozygosity in the human leukocyte antigen class I locus (HLA LOH) in the four major disease subtypes and identified largely similar patterns of HLA LOH across the different *KRASm* isoforms (Supplemental Fig. 7). In non-Sq NSCLC, 22% of G12C-mutated cases exhibited HLA LOH; in comparison 20% to 23% of the other isoforms and 21% of WT cases had HLA LOH. In PDAC, G12D mutated cases showed a higher prevalence of HLA LOH (28%) compared to G12V (23%, *p* = 0.03) and G12R mutated cases (22%, *p* = 0.04). Rate of HLA LOH was also slightly elevated in *KRAS* G12D mutated CRC (18%) compared to *KRAS*-WT CRC (15%, *p* = 0.008).

We also looked at mutational signatures across *KRAS*m isoforms of the four major disease subtypes (Fig. 4d and Supplemental Table 14). Tobacco signature was detected in 38–45% of different *KRAS*m and *KRAS* WT subsets of non-Sq NSCLC but was less frequent in G12R non-Sq NSCLC (22%) and rare in other tumor subtypes. Mismatch repair (MMR) signature was rare in non-Sq NSCLC but common in *KRAS*m and *KRAS* WT PDAC, CRC and endometrial cancers, although relatively less frequent in *KRAS* G12C CRC relative to other subgroups. POLE signature was detected in small subsets of PDAC, CRC, and endometrial.

We further assessed the co-occurrence of *KRAS*m isoforms and co-altered genes with implications for immunotherapy response including TMB ≥ 10 mutations/Mb, and PD-L1 expression in non-Sq NSCLC and Sq NSCLC for comparison. In non-Sq NSCLC, TMB and PD-L1 were independent biomarkers; across *KRAS*m isoforms 49–62% had TMB ≥ 10 mutations/Mb or high PD-L1, but only 15–20% had both. With *KRAS* G12C mutated non-Sq NSCLC samples, co-mutations in *STK11* and *KEAP1* were more commonly associated with low (28% and 16%) or negative (53% and 27%) PD-L1 expression vs high (9.4% and 8.3%), and these associations were largely consistent across *KRAS*m subsets. Across *KRAS*m isoforms of non-Sq NSCLC, 46–51% harbored *TP53*, 5.1–9.2% *TP53*/*STK11* and 1.3–2.6% *TP53*/*STK11*/*KEAP1* co-alterations (Supplemental Fig. 8). In non-Sq NSCLC, there were 1487 distinct mutations throughout the *TP53* gene in *KRAS*m tumors, where 45% resulted in single amino acid changes. Co-mutations occurred throughout both *STK11* and *KEAP1* genes in *KRAS*m tumors; *NEF2L2* co-mutations were generally uncommon and clustered around G31 and G81 positions (Supplemental Fig. 9).

### Clinico-genomic database outcomes analysis

A total of 16,357 patients with advanced NSCLC (aNSCLC), 10,430 patients with metastatic CRC (mCRC), and 3323 patients with mPDAC were included in the Flatiron Health-Foundation Medicine CGDB with 5938, 3838, and 1398 patients, respectively, meeting eligibility criteria, having started first line (1 L) of therapy after tissue biopsy CGP, and having available clinical characteristics for outcomes assessment (Supplemental Fig. 10).

**Fig. 5:**
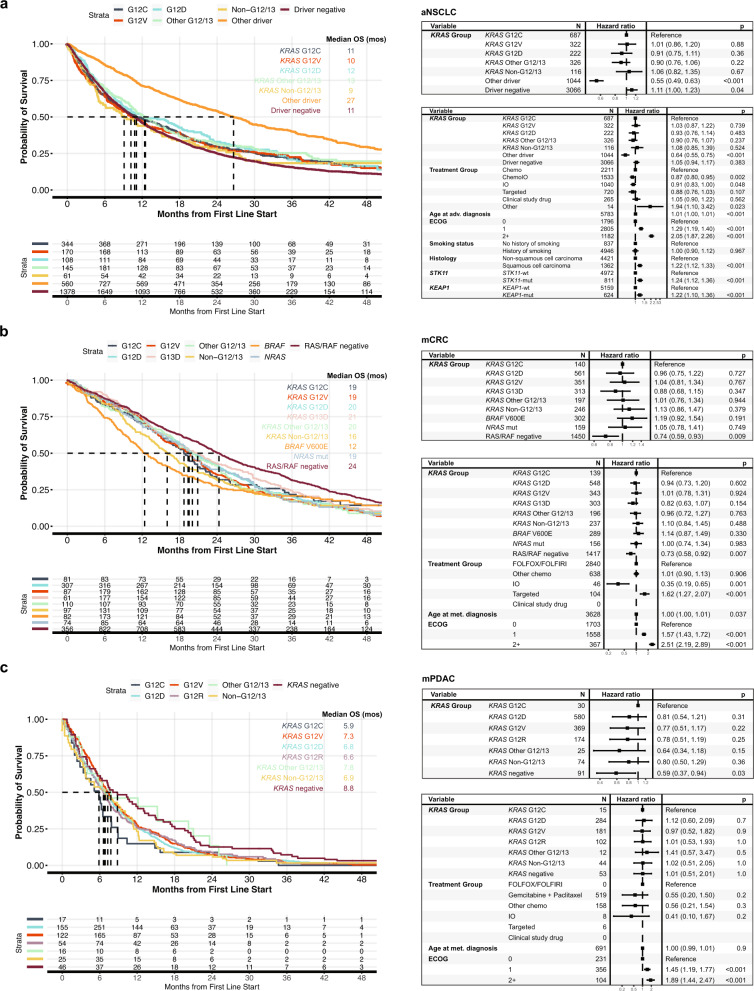
Real-world outcomes for patients with NSCLC, PDAC, and CRC carrying different oncodriver alterations. Kaplan Meier curves for real world overall survival (rwOS) are shown with univariate (top) and multivariate (bottom) analysis tables for each disease subtype. Analysis was performed using the Flatiron Health-Foundation Medicine real-world clinicogenomic database. Patients with multiple driver alterations spanning >1 category were excluded. **a** In patients with advanced NSCLC harboring *KRAS* G12C mutant tumors have similar rwOS to other *KRAS* G12/13, *KRAS* non-G12/G13C, and driver negative patients. **b** In metastatic CRC, patients with *KRAS* G12C had similar rwOS compared to other *KRAS* mutant isoforms, *BRAF* V600E, and *NRAS* mutations, but worse rwOS compared to patients negative for *KRAS* and *NRAS* mutations and *BRAF* V600E (*RAS/RAF* negative). **c** In metastatic PDAC, rwOS was marginally inferior for *KRAS* G12C vs *KRAS* WT, although the differences were small and were not observed in the multivariable model.

Among aNSCLC patients, *KRAS*m were identified in 29% of cases including 93% at codons G12 or G13 (G12/13) and 6.8% at non-G12/13 codons. Patients with *KRAS*m predominantly had non-Sq histology and most frequently were treated with 1 L chemotherapy or chemotherapy with immune checkpoint inhibitor (ICI), although over 20% of *KRAS*m patients received ICI monotherapy (Supplemental Table 15). Patients with *KRAS G12C* mutation had similar overall survival (OS) to patients with other common *KRAS* mutations including G12V (11 vs. 10 mos, HR 1.0, 95% CI 0.86–1.20, *p* = 0.88) and G12D (11 vs. 12 mos, HR 0.91, 95% CI 0.75–1.11, *p* = 0.36) and also similar OS with rarer non-G12/13 *KRAS*m (11 vs. 9 mos, HR 1.1, 95% CI 0.82–1.35, *p* = 0.67). OS for NSCLC oncodriver negative patients, although statistically different, there was very minimal clinical difference (11 vs 11 mos, HR 1.1, 95% CI 1.00–1.23, *p* = 0.04). Patients with aNSCLC without a *KRAS* alteration but exhibiting other oncogenic drivers with available approved targeted therapies, had significantly better OS than patients with NSCLC harboring *KRAS* G12C mutations (27 vs. 11 mos, HR 0.55, 95% CI 0.49–0.63, *p* < 0.001). These trends remained similar in a multivariable model incorporating treatment group, age, performance status, smoking history, histology, and concurrent *STK11* and *KEAP1* alterations (Fig. 5a).

Among mCRC patients, 49% had a *KRAS*m, 8.1% had *BRAF* V600E, 4.2% had an *NRAS*m, and 39% of cases were negative for *KRAS*m, *NRAS*m, and *BRAF* V600E (Supplemental Table 16).

Patients with *KRAS* G12C mutated tumors had similar OS to patients with other common *KRAS* mutations including G12D (19 vs. 20 mos, HR 0.96, 95% CI 0.75-1.22, *p* = 0.73) and G12V (19 vs. 19 mos, HR 1.04, 95% CI 0.81-1.34, *p* = 0.77). Patients with *KRAS* G12C mutated tumors had slightly elevated OS compared to patients with *BRAF* V600E (19 vs. 12 mos, HR 1.19, 95% CI 0.92-1.54, *p* = 0.19) and patients with non-G12/13 mutations (19 vs 16 mos, HR 1.13, 95% CI 0.86-1.47, *p* = 0.38), although not statistically significant. *KRAS*/*NRAS*/*BRAF* V600E negative (*RAS*/*RAF* negative) patients had more favorable OS than the *KRAS* G12C subgroup (24 vs. 19 mos, HR 0.74, 95% CI 0.59–0.93, *p* = 0.009), which was consistent in a multivariable model (Fig. 5b).

Finally, *KRAS*m were identified in 93% of patients with metastatic PDAC, 94% of which occurred within codons G12/G13 (Supplemental Table 17). Patients with tumors harboring *KRAS* G12C mutation had statistically worse, though clinically similar, OS compared those with *KRAS* negative tumors (5.9 vs. 8.8 mos, HR 0.59, 95% CI 0.37-0.94), *p* = 0.03) and similar OS to patients with non-G12/13 *KRAS*m tumors (5.9 vs. 6.9 mos, HR 0.80, 95% CI 0.50–1.29, *p* = 0.36). However, in a multivariable model, patients with *KRAS* G12C mutated tumors were not significantly associated with worse OS compared to patients with *KRAS* negative tumors, although this may be limited by the small number of *KRAS* negative cases (Fig. 5c).

## Discussion

We performed the largest pan-cancer survey to date of *KRAS* alterations in patients with solid tumors and hematologic malignancies. *KRAS* is a pan-tumor oncodriver with alterations identified in 23% of over 400,000 adult cases, including 21% with mutations, similar to smaller published studies^17–19^. In common tumor types including NSCLC, CRC and PDAC, *KRAS* mutation frequencies and hotspot mutation clusters were confirmatory of published literature^20,21^. Taking a disease focused approach, we observed that non-Sq NSCLC, CRC, and PDAC represented 71% of all patients with *KRAS*m cancers; the most common alterations pan-cancer were *G12D* (30%), *G12V* (23%) and *G12C* (15%), totaling 68% of *KRAS*m cases, which represent potential therapeutic targets. However, we also explored less well characterized *KRAS* landscapes such as in endometrial cancer, where *KRAS G12V* and *G12D* are predominant, as well as small bowel and appendiceal cancers, cholangiocarcinoma, and others. Across 24 major tumor types studied, *KRAS*m were detected at frequencies from 1.3% to 92%. We also observed *KRAS* alterations in 5.5% of over 7000 pediatric cases assessed, with G13D being the most common variant. In a sub analysis of CGP of liquid biopsies, we observed a similar frequency and distribution of *KRAS* alteration and mutation isoforms pan-tumor. *KRAS* amplification is also a potential therapeutic target being explored, and we saw notable enrichment of this alteration in esophageal adenocarcinomas.

In our analysis, we assessed the presence of co-alterations in over 300 genes across *KRAS*m isoforms for eight disease types and observed some degree of similarity across *KRAS*m isoforms, which tended to be distinct from the *KRAS* WT landscape. On the other hand, we did observe some *KRAS*m specific differences in the prevalence of co-occurring gene alterations, immunotherapy-associated biomarkers and across six mutational signatures. In NSCLC, there is clear evidence that the co-alteration landscape in *KRAS*m cancers is an important determinant of outcomes. Previously published exploratory analysis of KRAS G12C inhibitor trials suggests that co-alterations in *KEAP1* and *STK11* may modulate outcomes to KRAS targeted therapies, though additional studies assessing these biomarkers are warranted^7,8,22^. *STK11* and *KEAP1*/*NEF2L2* have also been identified as negative predictors of outcomes to chemotherapy and immunotherapy, which remain important factors to consider when treating *KRAS*m NSCLC^23–25^. Our results confirm prior findings showing *STK11* and *KEAP1* alterations each occur more frequently in *KRAS* altered vs WT tumors, whereas driver alterations across disease types tend to be mutually exclusive with *KRAS*. Of note, our findings of elevated co-alterations in MAPK/PI3K pathway genes among *KRAS* G13D mutated CRCs and endometrial cancers compared to G12D mutated tumors, further supports that *KRAS* G13D mutated tumors may present unique biochemical and clinical mechanisms^26–28^. Overall, while we observed statistically significant differences in prevalence of some co-alterations, additional studies are warranted to examine the biological implications of these findings.

It is well established in NSCLC, CRC and other solid tumors that responses to KRAS inhibitors are variable; combination strategies in development will depend on characterization of the diverse genomic landscape of *KRAS* mutant cancers both pre-treatment and upon acquired resistance to therapies^29–31^. The significance of co-alterations, PD-L1 expression and mutational signatures in NSCLC and other *KRAS*m tumor types as prognostic and predictive markers for targeted therapies and immunotherapies, warrant future investigations. Numerous combination trials with KRAS and other MAPK pathway (SHP2/MEK/ERK) inhibitors are in progress aimed at improving outcomes in patients with *KRAS*m cancers and exploiting the presence of targetable co-alterations. Notably, in NSCLC and other solid tumors, several KRAS G12C inhibitors are being combined with immune checkpoint inhibitors (ICI), SHP2 inhibitors, EGFR inhibitors, and bevacizumab in clinical trials^22,29^. In CRC, trials of KRAS G12C inhibitors in combination with anti-EGFR therapies have shown promising initial results with 100% disease control rate reported for the combination of adagrasib and cetuximab^32^. With recent evidence for tumors employing loss of heterozygosity of the HLA locus as a common mechanism of immune evasion^33–35^, our study also presented the incidence of HLA LOH across cancers with frequent *KRAS* alterations. Investigating the role of tumor HLA status in the context of the clinical development of vaccines targeting *KRAS* mutations will play an important role in addressing biomarkers associated with clinical benefit from such therapies.

This study is limited by lack of available clinical and treatment information for patients in the Foundation Medicine genomic database not included in the CGDB. In the CGDB, clinical data were derived from EHR and may be incomplete or missing, particularly for events occurring outside of the Flatiron Health network. Finally, all patients in this study received CGP, which likely introduces selection bias. Our genomic analysis employs tumor-only sequencing (without matched normals) and subsequent filtering is relied on to select variants known or likely to be pathogenic for inclusion in analysis.

Notwithstanding these limitations, the findings from this study have significant implications for the development of KRAS inhibitors targeting G12C, G12D, G12V and beyond. Genomic profiling to detect co-alterations and mutational signatures, and trials to understand the clinical importance of these biomarkers as predictors of response to targeted therapies and immunotherapies in patients with a wide range of *KRAS* altered tumor types will be imperative to improve therapy selection and outcomes.

## Methods

### Foundation medicine comprehensive genomic profiling

We interrogated pan-cancer cases submitted for comprehensive genomic profiling (CGP) during routine clinical care (Foundation Medicine Inc., Cambridge, MA). For tissue biopsy samples, DNA was extracted from 40 microns of FFPE sections, and CGP was performed on hybridization-captured, adapter ligation based libraries to a mean coverage depth of >550X for 315 (*n* = 143,020), 324 (*n* = 250,197) or 405 (*n* = 40,730) cancer-related genes and selected introns from 28, 31, or 36 genes frequently rearranged in cancer^36^ (Supplemental Table 18). TMB was calculated by counting the number of non-driver synonymous and non-synonymous mutations across a 0.8–1.2 megabase (Mb) region, with computational germline status filtering, and reporting as mutations/Mb. This method has been previously validated for accuracy against whole exome sequencing^37^. Microsatellite instability (MSI) was determined by analyzing intronic homopolymer repeat loci for length variability and compiled into an overall MSI score via principal component analysis^38^. Results were analyzed for substitutions, short insertions/deletions and rearrangements, and copy number changes. A statistical copy number model was generated and fitted to each sample to determine gene copy numbers. *KRAS* amplifications as described here include amplifications ≥4 copies above the overall ploidy of the specimen. In most cases, this represents amplifications ≥6 copies. The detection of gene alterations followed a multi-step approach, as described previously^36,39,40^. Briefly, commonly occurring germline variants were excluded based on their presence in public databases such as dbSNP, 1000 Genomes Project and ExAC^41–43^; however, known pathogenic germline variants (e.g., in *BRCA1*, *BRCA2*) were considered as reportable. Genomic alterations were designated as known or likely pathogenic using annotations such as presence in the COSMIC database, additional knowledge about the gene affected (e.g., truncations and deletions in known tumor suppressor genes), or mutations that have been characterized as pathogenic in the scientific literature; all other uncharacterized short variant alterations were denoted as variants of unknown significance (VUS) (Supplemental Fig. 11). Detected copy number alterations (amplifications of oncogenes and homozygous deletions of tumor suppressors) recurrent fusions or rearrangements (predicted to activate oncogenes or inactivate tumor suppressors) were also designed as known or likely to be pathogenic. For the analysis outlined in this study, only genomic alterations known or likely to be pathogenic were included and VUS were excluded.

Analysis of co-occurring and mutually exclusive gene alterations with *KRAS* alterations was limited to genes altered in at least 50 cases and targeted across all the assay versions. For each gene, substitutions, short insertions/deletions, rearrangements, and copy number changes of known or likely functional significance detected using our assay were included. A Fisher’s exact test with FDR-based correction for multiple testing was applied for this co-mutation analysis.

### Liquid CGP

For blood samples, cell free DNA (cfDNA) was extracted from blood plasma to create adapted sequencing libraries before hybrid capture and sample-multiplexed sequencing to a median unique exon coverage depth of >6000x for 62, 70, or 324 genes (Supplemental Table 18)^40^. Testing was performed in a CLIA-certified/CAP-accredited laboratory (Foundation Medicine Inc., Cambridge, MA).

The levels of ctDNA shed for each specimen was quantified by calculating an investigational composite tumor fraction (TF)^44^, which merges two methods for estimation of TF^45^. When TF is elevated (generally above 10%), an estimate is returned based on measure of tumor aneuploidy that incorporates observed deviations in coverage across the genome^46^. This aneuploidy-based approach avoids erroneously inferring elevated TF due to the presence of germline variants detected at high variant allele frequency. When lack of tumor aneuploidy limits the ability to estimate TF (generally at lower TF) a variant-based calculation is made by identifying the highest allele fraction non-germline variant, excluding specific clonal hematopoiesis (CH) associated alterations.

### PD-L1 expression

PD-L1 expression was determined by immunohistochemistry (IHC) performed on FFPE tissue sections in a CLIA certified/CAP-accredited laboratory using the Dako 22C3 PD-L1 antibody. A pathologist determined the percentage of tumor cells with expression (0–100%) and the intensity of expression (0, 1+, 2+). PD-L1 expression was reported as a continuous variable with the percentage of tumor cells staining with ≥1+ intensity. PD-L1 expression was summarized as negative (<1%), low positive (1–49%), or high positive (≥50% of tumor cells staining with ≥1+ intensity). IHC staining for PD-L1 was performed with Dako 22C3 antibody (catalog number SK006) according to the manufacturer’s instructions.

### Foundation Medicine-Flatiron Health Clinico-Genomic Database

We leveraged real-world data from the Flatiron Health (FH)-Foundation Medicine (FM) Clinico-Genomic Database (CGDB), a nationwide de-identified electronic health record (EHR)-derived database which includes patients sequenced at FM who received care within the FH network. The de-identified data originated from approximately 280 US cancer clinics (~800 sites of care). The FH-FM CGDB includes 16,357 patients with a diagnosis of advanced NSCLC, 10,430 with metastatic CRC, and 4438 with metastatic PDAC who received care within the FH network between 01/2011-09/2021. Cohorts included in our analysis were limited to those who had tissue CGP (FoundationOne^®^ or FoundationOne^®^CDx). Patients who were diagnosed with metastatic disease greater than 90 days prior to their first visit within the FH network or received their FMI report greater than 60 days after their last FH visit date were excluded to ensure all therapies received prior to CGP were captured. Additionally, patients who had their biopsy collected after starting first line (1 L) treatment were excluded to ensure all therapies received prior to CGP were captured and that the tumor genomics were accurate prior to 1 L treatment initiation. Retrospective longitudinal clinical data were derived from EHR data, comprising patient-level structured and unstructured data, curated via technology-enabled abstraction of clinical notes and radiology/pathology reports and linked to CGP data by de-identified, deterministic matching^47^.

### Mutational signatures

Mutational signatures for each sample were determined by examining the distribution of point mutations in the following six substitution classes: C > A, C > G, C > T, T > A, T > C, T > G, and their trinucleotide context from the bases flanking the mutated base, producing 96 possible combinations^48^. All point mutations were included in the analysis after excluding known oncogenic driver mutations and predicted germline mutations. In samples with at least 20 assessable mutations, the composition of the following six major mutational signatures was determined: Alkylating, APOBEC, MMR, POLE, Tobacco, and UV. A sample was deemed to have a dominant signature if a mutational class harbored a score of 0.4 or greater. Cases where a single dominant signature could not be identified were annotated as ‘None’.

### Estimation of clonality

The somatic-germline-zygosity (SGZ) algorithm was applied to each sample to distinguish somatic^49^. For each predicted somatic alteration observed in a sample, a tumor fraction was estimated from the variant allele fraction (AF), mutant copies (mc) and wild-type copies (wc), using the following formula: 2AF/(mc−AF(wc + mc-2)). The highest estimated tumor fraction from all the somatic alterations in a sample was used as an approximate for the tumor fraction of the sample. Clonal fraction of each alteration is obtained as the ratio of the variant and sample estimated tumor fractions, with ≥50% considered clonal. Samples that failed pipeline quality control thresholds and for which SGZ could not be run were excluded from this analysis.

### Determination of HLA class I loss of heterozygosity

Determination of HLA loss of heterozygosity was performed for samples profiled using FoundationOne® or FoundationOne® Heme assays^34^. Briefly, the minor allele frequency (MAF) of each HLA-I gene (HLA-A, HLA-B, and HLA-C) was calculated separately. HLA-I genotyping was performed using OptiType to a four-digit resolution^50^ and HLA-I reference sequences that matched the germline alleles for each sample were obtained. Only germline heterozygous alleles were assessed for LOH using the SGZ algorithm^49^.

### Statistical considerations

For analysis of clinical outcomes, real world overall survival (rwOS) was defined as time from first therapy administration to date of death^51^. Patients without a death event were censored at their date of last known activity. To account for left truncation and to reflect the process of cohort eligibility, a patient’s entry date into the clinicogenomic database (CGDB) was considered the later of the date of a patient’s second visit within the FH network or their first eligible FM CGP report. Risk set adjustment was used to ensure patients treated prior to entry date were not included in the at-risk population in OS analysis until they reached their entry date.

### IRB approval

For Foundation Medicine (FMI) genomic analysis, approval for this study, including a waiver of informed consent and a HIPAA waiver of authorization, was obtained from the WCG Institutional Review Board (IRB; Protocol No. 20152817). The IRB granted a waiver of informed consent under 45 CFR § 46.116 based on review and determination that this research meets the following requirements: (i) the research involves no more than minimal risk to the subjects; (ii) the research could not practicably be carried out without the requested waiver; (iii) the waiver will not adversely affect the rights and welfare of the subjects. The CGDB is a de-identified database, where data from Foundation Medicine and Flatiron are linked by an independent third party and there is no route to identify the included patients. For the Flatiron Health-Foundation Medicine CGDB analysis, IRB approval with waiver of informed consent based on the observational, non-interventional nature of the study (WCG IRB, Protocol No. 420180044) was also obtained prior to study conduct.

## Supplementary information

Supplemental figures

Supplemental tables

## Data Availability

The authors declare that all relevant aggregate data supporting the findings of this study are available within the article and its supplementary information files. In accordance with the Health Insurance Portability and Accountability Act, we do not have IRB approval or patient consent to share individualized patient genomic data, which contains potentially identifying or sensitive patient information and cannot be reported in a public data repository. Foundation Medicine is committed to collaborative data analysis and has well established and widely used mechanisms by which qualified researchers can query our core genomic database of >500,000 de-identified sequenced cancers. More information and mechanisms for data access can be obtained by contacting the corresponding author or the Foundation Medicine Data Governance Council at data.governance.council@foundationmedicine.com.

## Code availability

This study made use of publicly available packages with R version 4.0.3 and Python 2.7. Code used to generate figures are available upon request.
