# Supplementary material for: Comprehensive pan-cancer genomic landscape of KRAS altered cancers and real-world outcomes in solid tumors
Source: NPJ Precis Oncol. 2022 Dec 9;6:91. doi: 10.1038/s41698-022-00334-z (PMC9734185; doi:10.1038/s41698-022-00334-z)
Supplement: Supplementary file 1 — Supplemental figures [file 41698_2022_334_MOESM1_ESM.pdf]

Supplemental Figure 1. Clonality of *KRAS* mutation subtypes across 4 major tumor types.

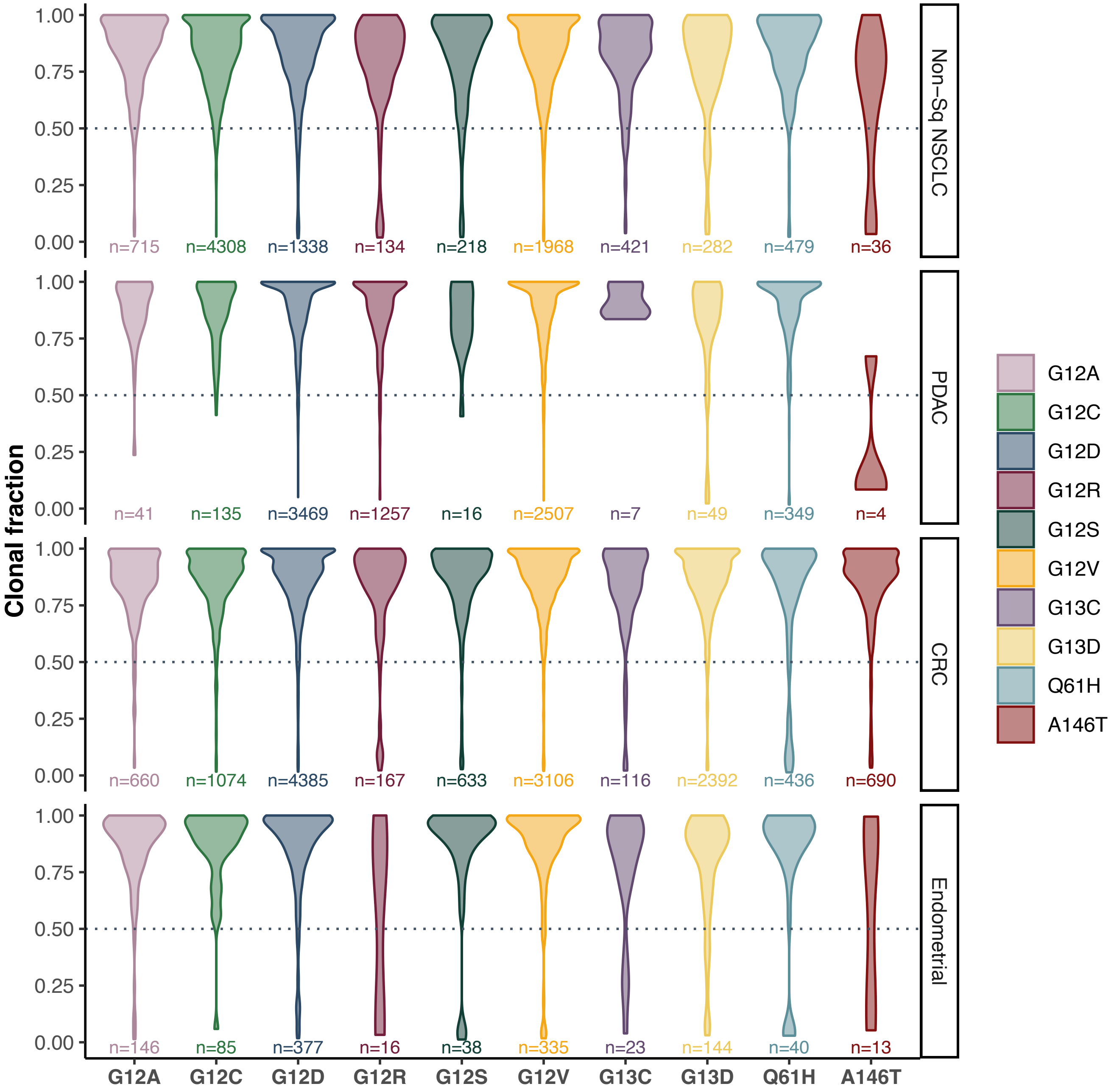

Violin plots showing the distribution of clonal fraction for different *KRAS* mutations in non-Sq NSCLC, PDAC, CRC, and endometrial tumors. Color corresponds to the *KRAS* mutation. A dashed line at a clonal fraction of 0.5 (50%) is shown. A vast majority of *KRAS* mutations are identified to be clonal.

Supplemental Figure 2. *KRAS* alteration frequencies comparing tissue and liquid CGP.

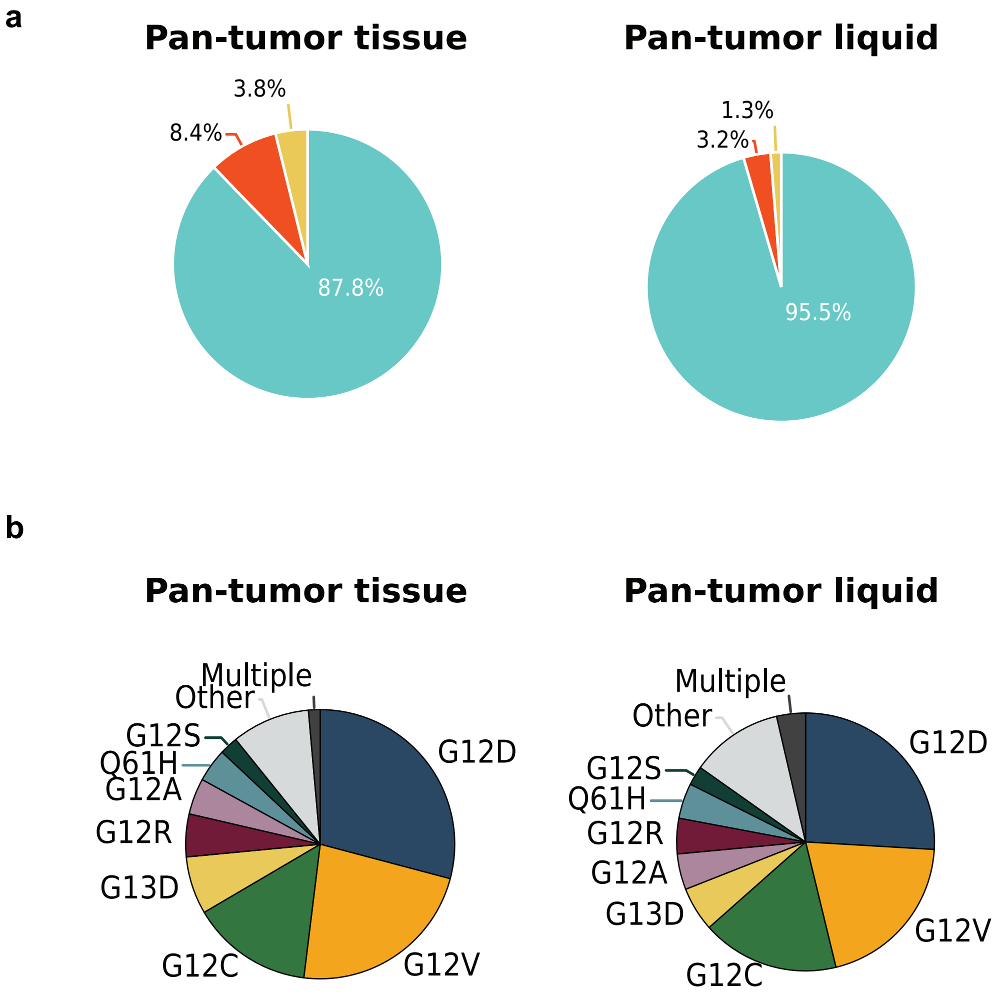

KRAS alteration types: green (mutation), red (amplification) or yellow (both) (a) and mutation subtypes (b) were detected at similar prevalence in tissue (n= 426,706) and liquid (n= 62,369) biopsy cohorts using CGP during routine clinical care.

Supplemental Figure 3. Prevalence of *KRAS* alterations across pediatric cancers.

a

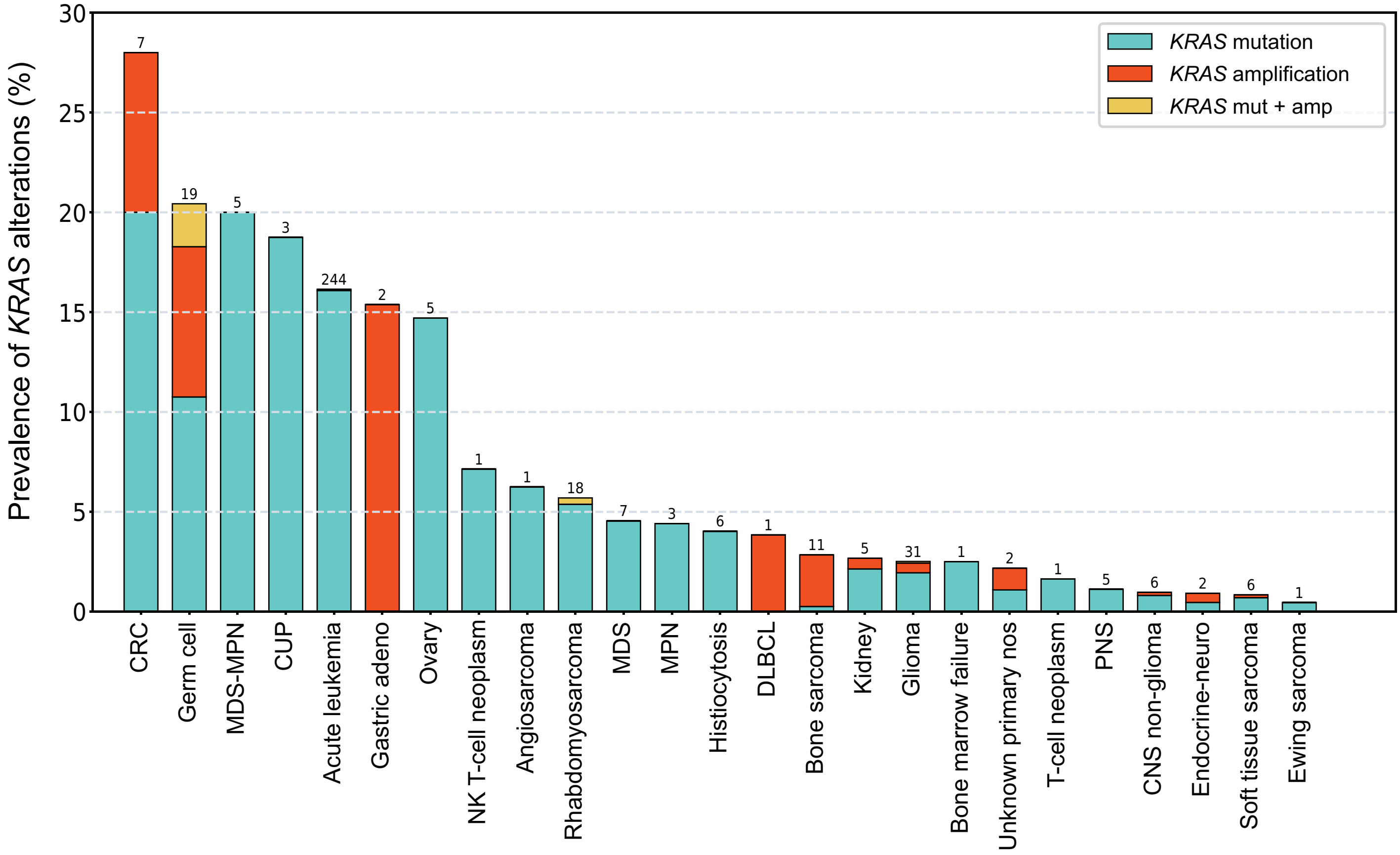

b

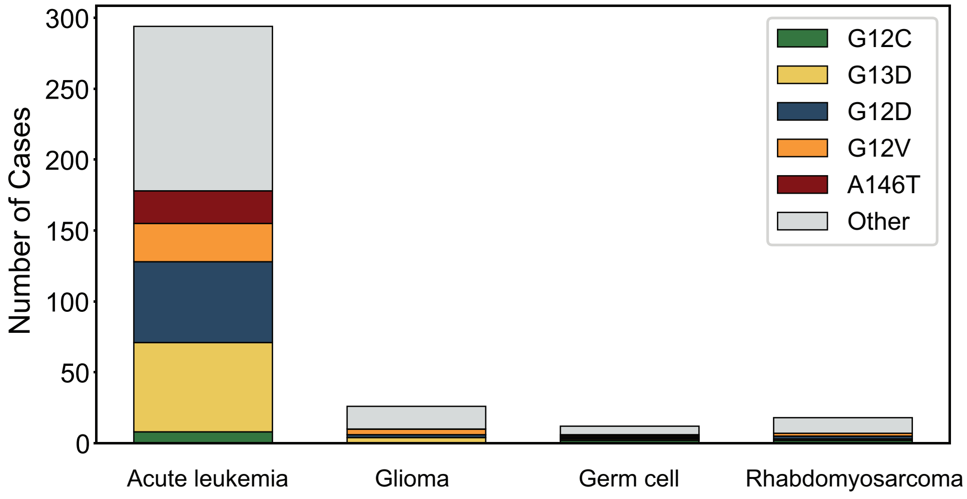

(a) Prevalence in the Foundation Medicine (FM) dataset of tissue of hematologic samples from 7,241 pediatric patients with cancer. *KRAS* mutations are most prevalent in pediatric CRC, MDS-MPN, carcinoma of unknown primary (CUP) and acute leukemia cancer types. (b) Incidence of potential intend to treat pediatric populations in the FM dataset based on the 4 largest *KRAS* mutant disease subtypes. Acute leukemia represents by far the largest pediatric *KRAS* mutant population and includes diverse *KRAS* mutation subtypes.

Supplemental Figure 4. Genes enriched for alterations co-occurring and mutually exclusive with *KRAS* alterations in microsatellite stable colorectal and endometrial cancer.

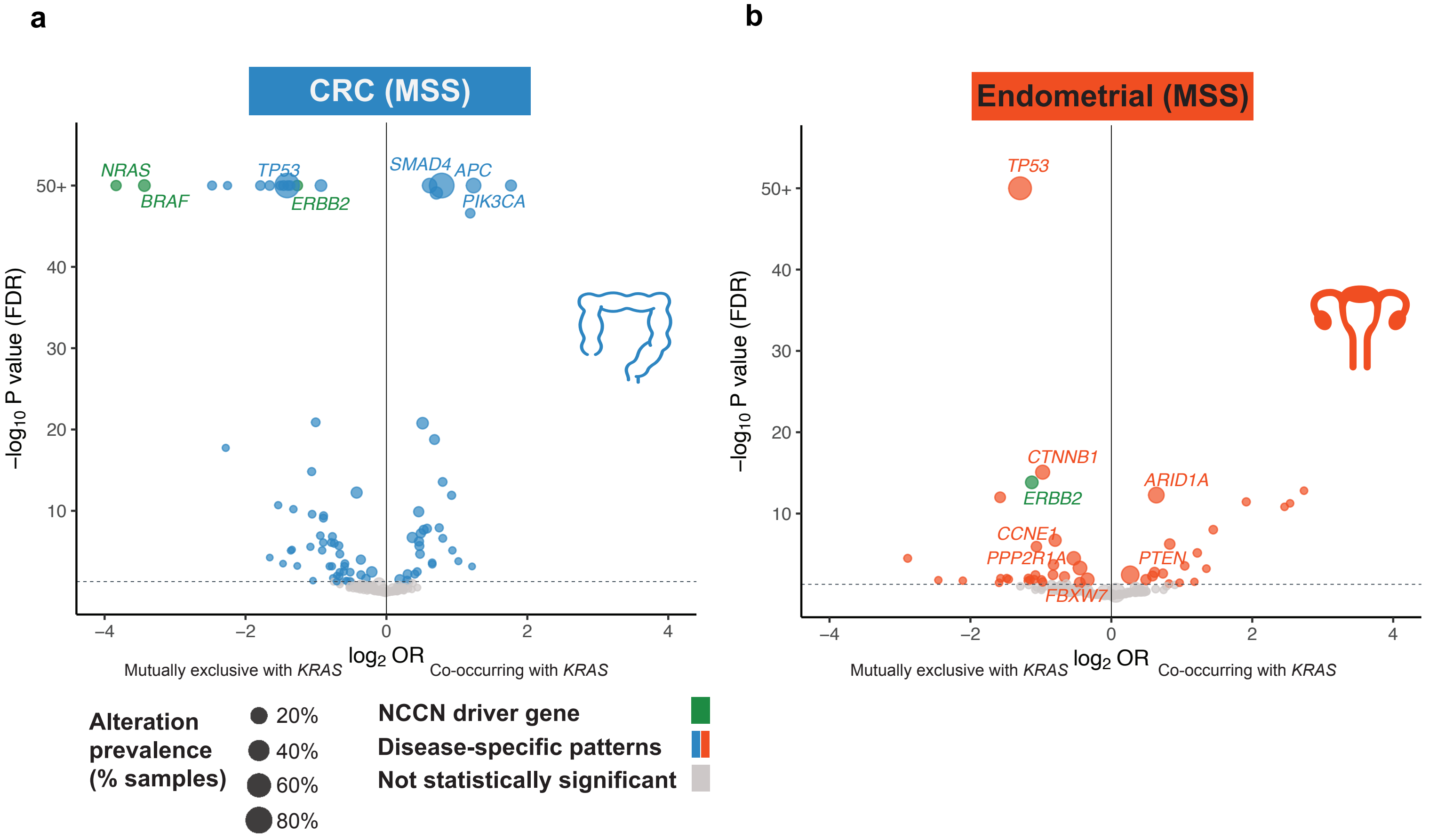

Specimens with alterations in known driver genes (labeled in green) are enriched for mutual exclusivity with *KRAS* alterations (left side of plots) in microsatellite stable (MSS) (a) colorectal (CRC; N = 44,859) and (b) endometrial (N = 11,592) cancers. Overall, patterns of co-occurrence were similar to the overall cohort within these tumor types, outlined in Figure 3. Fisher's exact test was applied to assess patterns of co-occurrence and mutual exclusivity between *KRAS* and other genes alterations. P values were corrected with the Benjamini-Hochberg FDR method. Driver genes highlighted in the National Comprehensive Cancer Network (NCCN) Guidelines as well as high prevalence genes (≥10%) are labeled for each volcano plot.

Supplemental Figure 5. Genes enriched for alterations co-occurring and mutually exclusive with *KRAS* alterations.

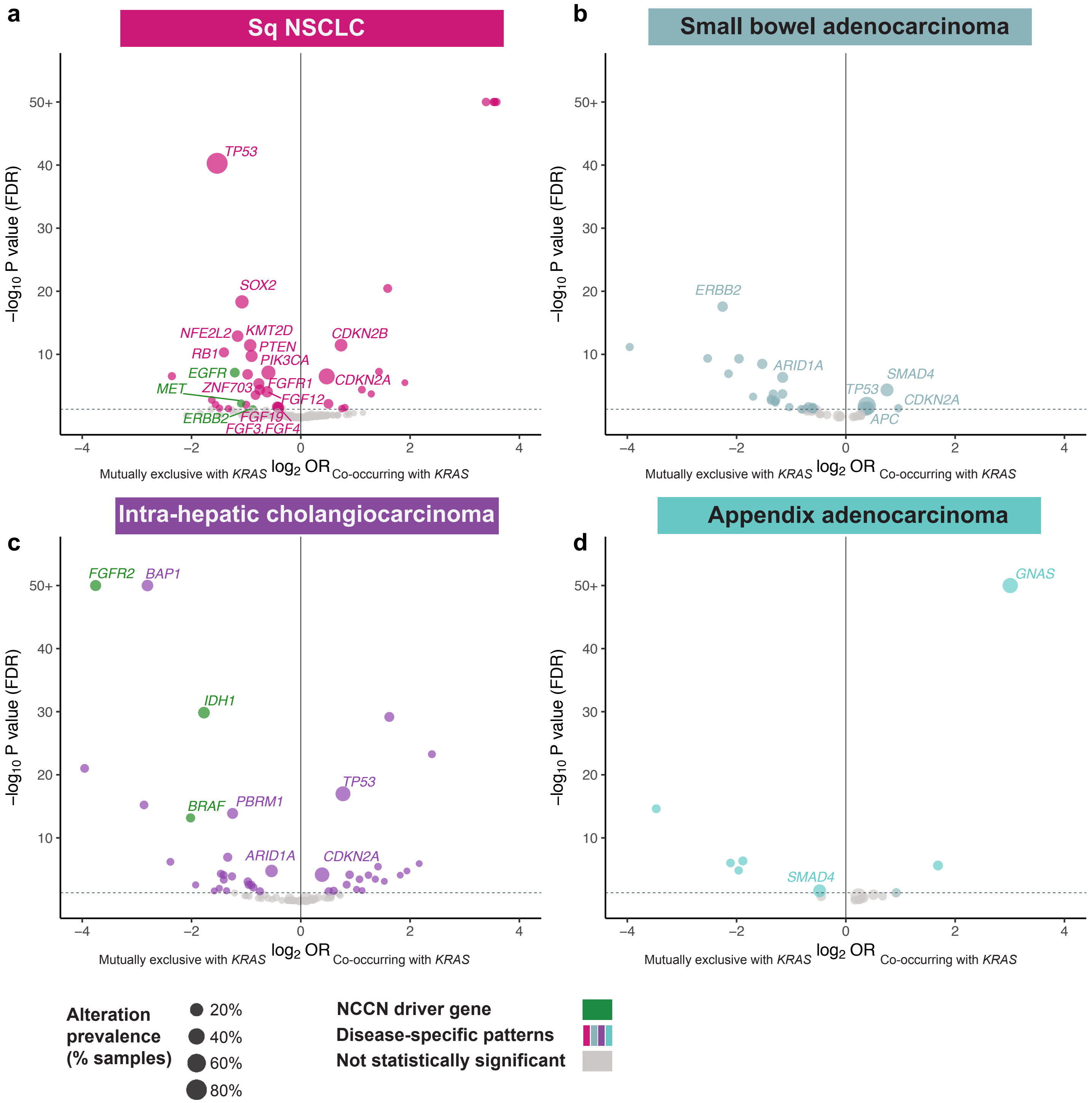

(a-d) The prevalence of alterations between *KRAS*-altered and *KRAS* wild type (WT) sq NSCLC (N = 15,499), small bowel adenocarcinoma (N=1,859), intra-hepatic cholangiocarcinoma (N=7,467), and appendix adenocarcinoma (N=1,526). Specimens with alterations in known driver genes (labeled in green) are enriched for mutual exclusivity with *KRAS* alterations (left side of plots) in all four tumor types studied. Other genes with high alteration prevalence that tend to co-occur with *KRAS* alterations are *TP53* and *STK11* in intrahepatic cholangiocarcinoma (c) and *GNAS* in appendix adenocarcinoma (d). Fisher's exact test was applied to assess patterns of co-occurrence and mutual exclusivity between *KRAS* and other genes alterations. P values were corrected with the Benjamini-Hochberg FDR method. Driver genes highlighted in the National Comprehensive Cancer Network (NCCN) Guidelines as well as high prevalence genes (≥10%) are labeled for each volcano plot.

Supplemental Figure 6. Co-occurrence of select genes by *KRAS* mutation subtype for the 4 major tumor types.

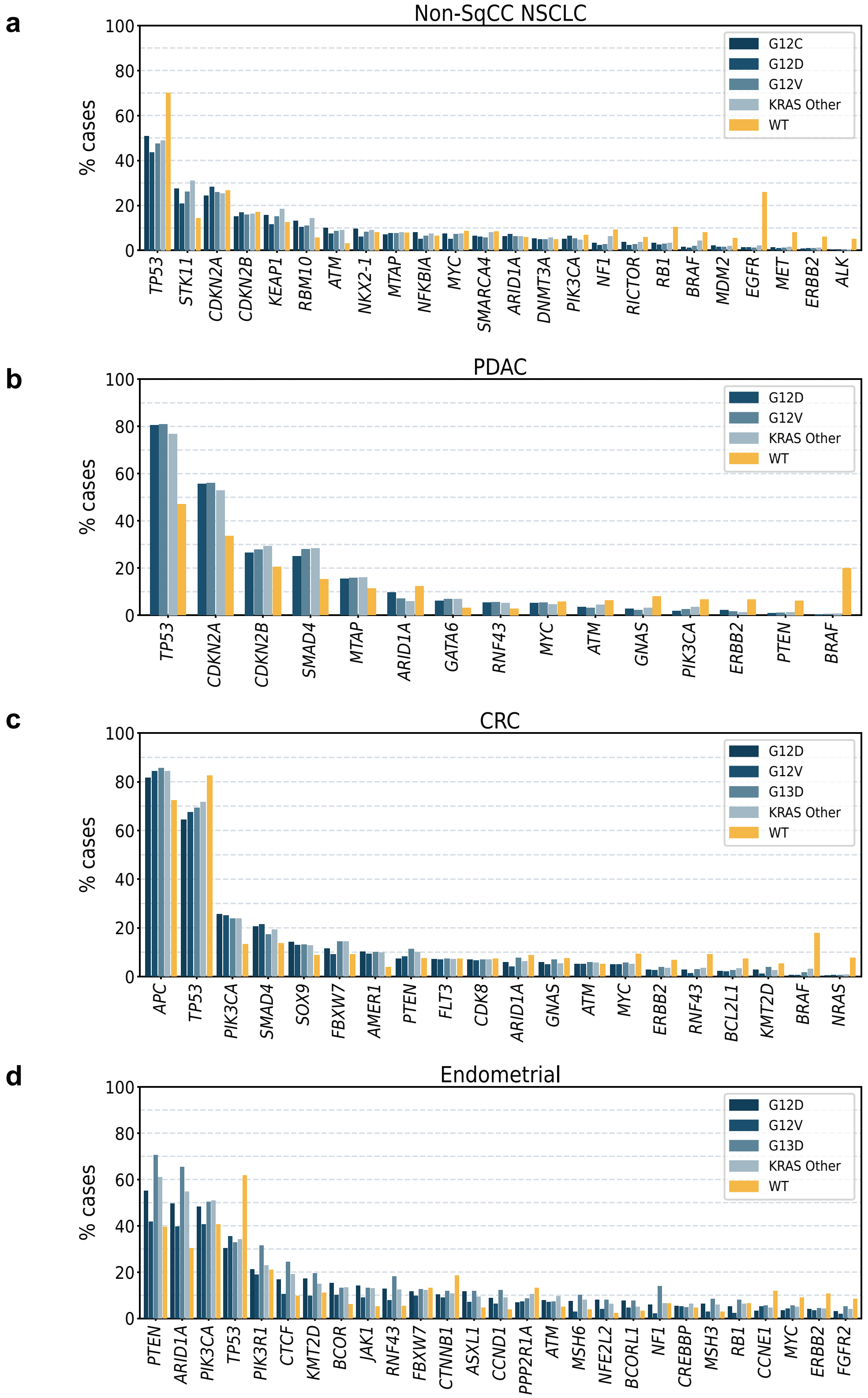

Longtail plots of co-altered genes with common *KRAS* mutation subtypes for (a) non-Sq NSCLC (b) PDAC (c) CRC and (d) endometrial cancer. Included genes are all genes altered in at least 5% of *KRAS* WT or *KRAS* mutant samples. *KRAS* G12C was assessed for Non-Sq NSCLC only, due to low prevalence in other tumor types. *KRAS* G13D was assessed in CRC and endometrial due to its higher prevalence in those tumor types. Co-alteration frequencies were largely similar across *KRAS* mutation subtypes but distinct from *KRAS* WT.

Supplemental Figure 7. Patterns of HLA loss of heterozygosity in the major *KRAS*-altered tumors

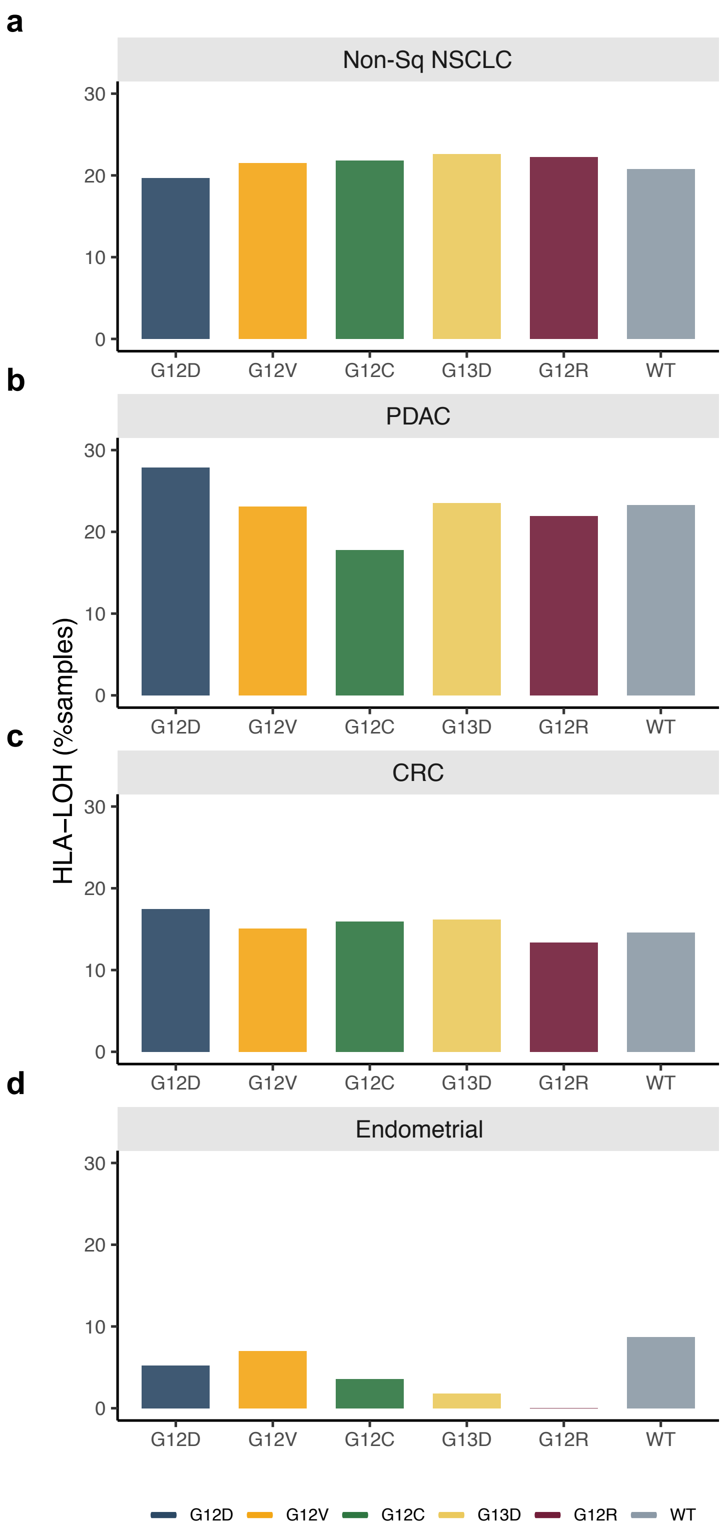

	G12D	G12V	G12C	G13D	G12R	WT
N_{total}	514	666	1,538	93	45	7,951
$N_{HLA\text{-}LOH}$	101	143	335	21	10	1,653
$\%_{HLA\text{-}LOH}$	19.6	21.5	21.8	22.6	22.2	20.8

	G12D	G12V	G12C	G13D	G12R	WT
N_{total}	930	675	45	17	342	271
$N_{HLA\text{-}LOH}$	259	156	8	4	75	63
$\%_{HLA\text{-}LOH}$	27.8	23.1	17.8	23.5	21.9	23.2

	G12D	G12V	G12C	G13D	G12R	WT
N_{total}	1,477	1,029	389	786	60	4,986
$N_{HLA\text{-}LOH}$	258	155	62	127	8	728
$\%_{HLA\text{-}LOH}$	17.5	15.1	15.9	16.2	13.3	14.6

	G12D	G12V	G12C	G13D	G12R	WT
N_{total}	134	143	28	56	3	2,198
$N_{HLA\text{-}LOH}$	7	10	1	1	0	192
$\%_{HLA\text{-}LOH}$	5.2	7.0	3.6	1.8	0.0	8.7

Prevalence of HLA loss of heterozygosity (LOH) in (a) non-Sq NSCLC (b) PDAC (c) CRC and (d) endometrial cancer. Rates of HLA LOH were largely similar across *KRAS* mutation subtypes and *KRAS* wildtype (WT) groups, although some moderate differences were observed in specific *KRASm* isoforms in CRC and PDAC.

Supplemental Figure 8. Co-occurrence patterns of immunotherapy biomarkers in NSCLC.

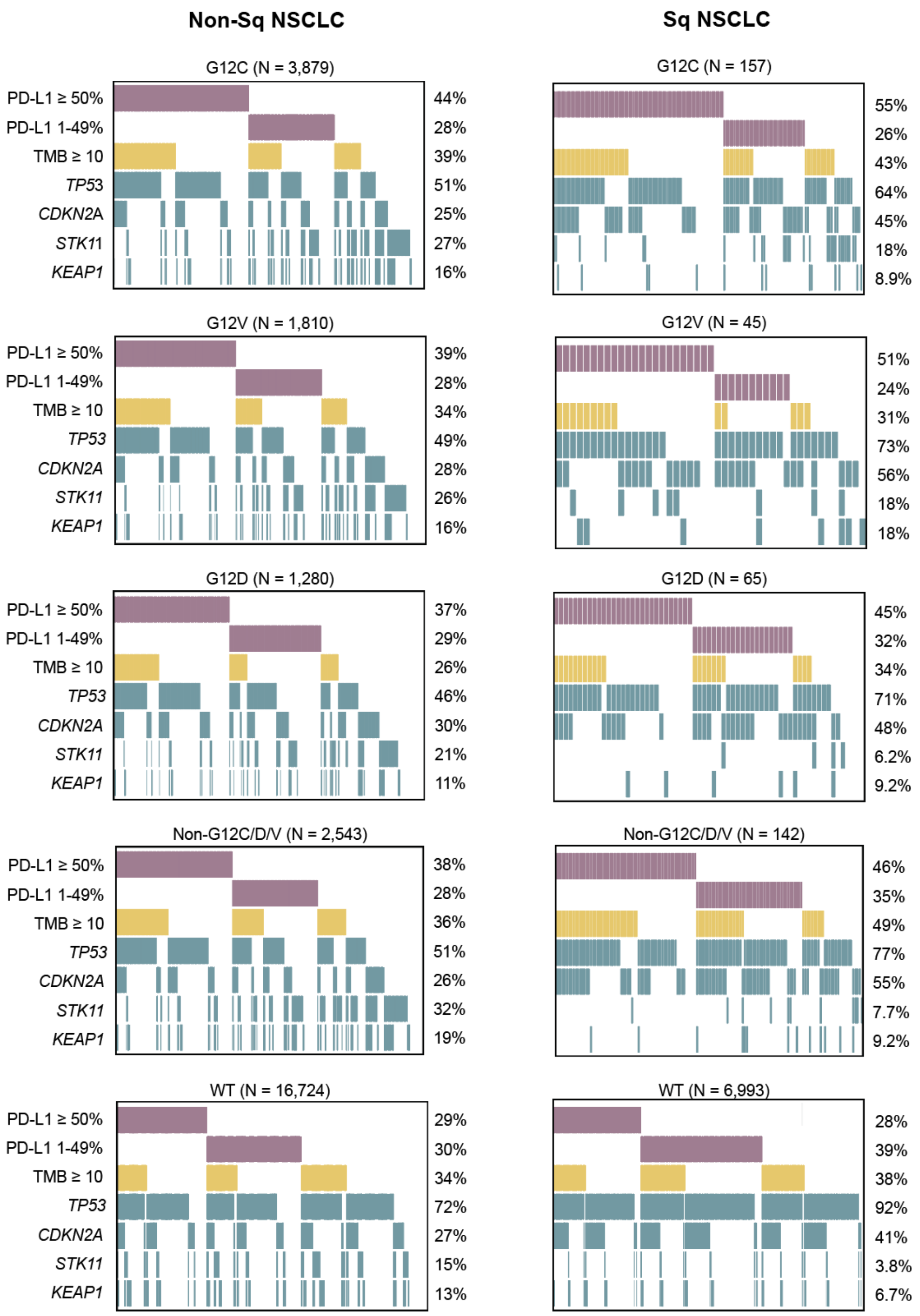

Oncoprints showing co-occurrence of immunotherapy biomarkers in *KRAS* mutant WT subsets of non-Sq NSCLC (left) and Sq NSCLC (right). This analysis is limited to 26,236 non-Sq NSCLC samples and 7,402 Sq NSCLC samples with PD-L1 IHC data available. PD-L1 and TMB were largely independent biomarkers with only 15-20% of non-Sq NSCLC samples having both high PD-L1 and TMB ≥10 mutations/Mb depending on the *KRAS* mutation subtype. *STK11* and *KEAP1* were more commonly associated with low or negative PD-L1 expression vs high and these associations were largely consistent across *KRAS* mutation subsets.

Supplemental Figure 9. Visualization of mutation types and positions across *TP53*, *STK11* , *KEAP1* and *NFE2L2* genes in non-Sq NSCLC.

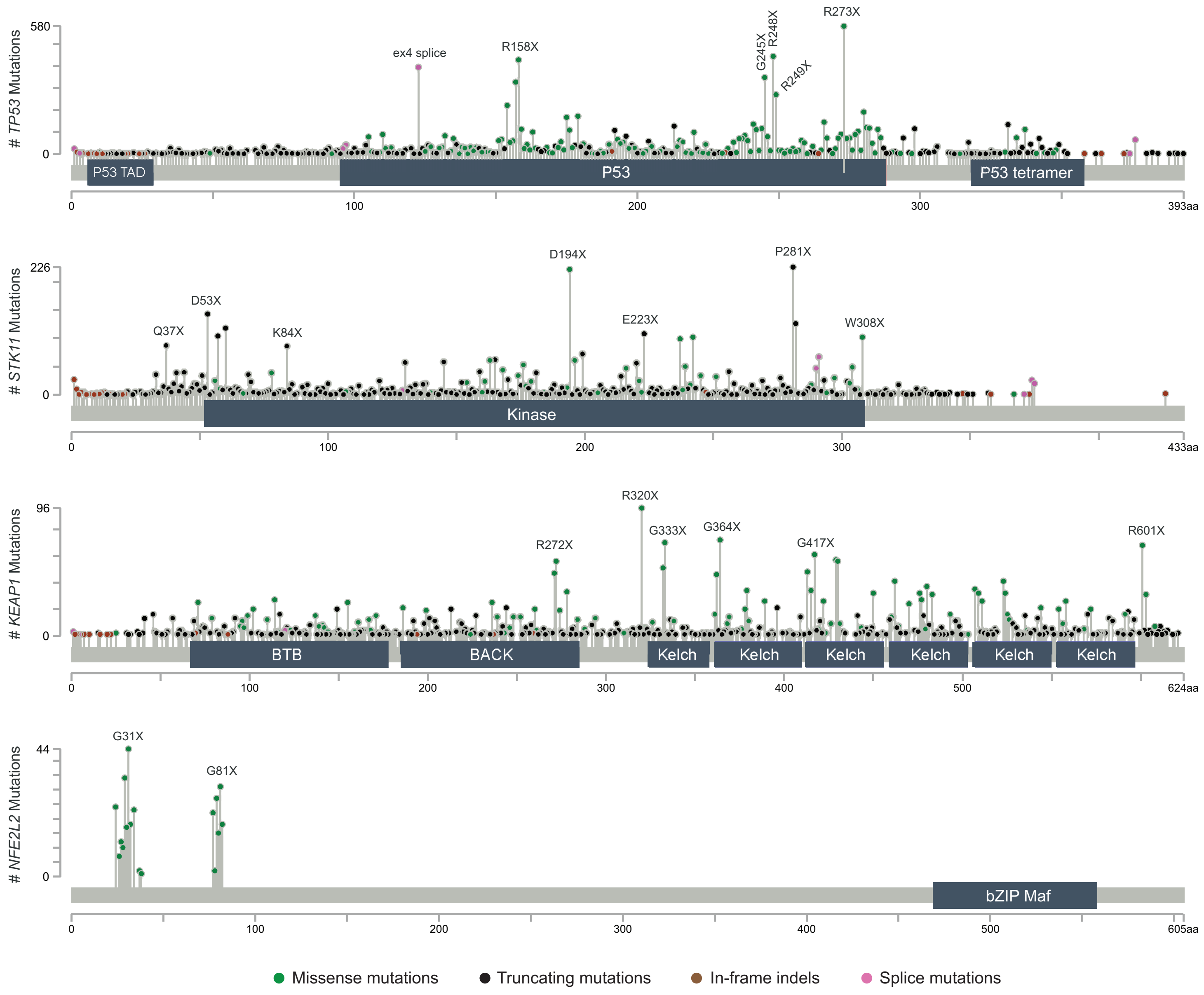

Lollipop plots showing positions of mutations with known or likely functional significance in each of 4 genes for non-Sq NSCLC samples. Dot shape and color represents different mutation types. *TP53*, *STK11* and *KEAP1* co-mutations were very diverse; *NEF2L2* co-mutations were generally uncommon and clustered around G31 and G81 positions.

Supplemental Figure 10. Consort diagram for CGDB analyses.

	NSCLC	CRC	PDAC
Total patients	16,357	10,430	3,323
Adv/met diagnosis after 1/2011, CGP within 60 days of last FH visit, structured activity within 90 days of adv/met diagnosis	12,152	6,969	2,379
Received tissue CGP	9,275	6,286	2,117
Specimen collection before 1L therapy start	6,745	4,548	1,576
Available disease-specific clinical characteristics*	5,938	3,838	1,398
Driver status	*KRAS* G12C 701<br>*KRAS* G12V 330<br>*KRAS* G12D 224<br>*KRAS* other G12/13 337<br>*KRAS* non-G12/13 118<br>Other NSCLC driver 1,055<br>Multiple drivers 34<br>Driver-neg 3,139	*KRAS* G12C 143<br>*KRAS* G12D 570<br>*KRAS* G12V 360<br>*KRAS* G13D 319<br>*KRAS* other G12/13 203<br>*KRAS* non-G12/13 254<br>*BRAF* V600E 311<br>*NRAS* mut 160<br>Multiple RAS/RAF 34<br>RAS/RAF neg 1,484	*KRAS* G12C 31<br>*KRAS* G12D 605<br>*KRAS* G12V 380<br>*KRAS* G12R 181<br>*KRAS* other G12/13 25<br>*KRAS* non-G12/13 76<br>Multiple *KRAS* 4<br>*KRAS-neg* 96

Consort diagram for Figure 5 analyses performed on cohorts from the Foundation Medicine- Flatiron Health clinic-genomic database (CGDB). Cohort eligibility diagram. For NSCLC patients, "Other driver" refers to activating *ALK/RET/ROS1* rearrangements, *BRAF* V600E, *EGFR* L858R/exon 19 deletion/S768I/L861Q/G719X, *MET* exon 14 skipping alterations or *NTRK* fusions. For CRC, "RAS/RAF neg" refer to patients with tumors negative for *KRAS* and *NRAS* known or likely pathogenic mutations and *BRAF* V600E. *Disease-specific clinical characteristics refer to ECOG for all three tumor types, and additionally histology and smoking status for NSCLC.

Supplemental Figure 11. Flow chart for inclusion of short variant alterations of known or likely pathogenicity in co-mutation analyses.

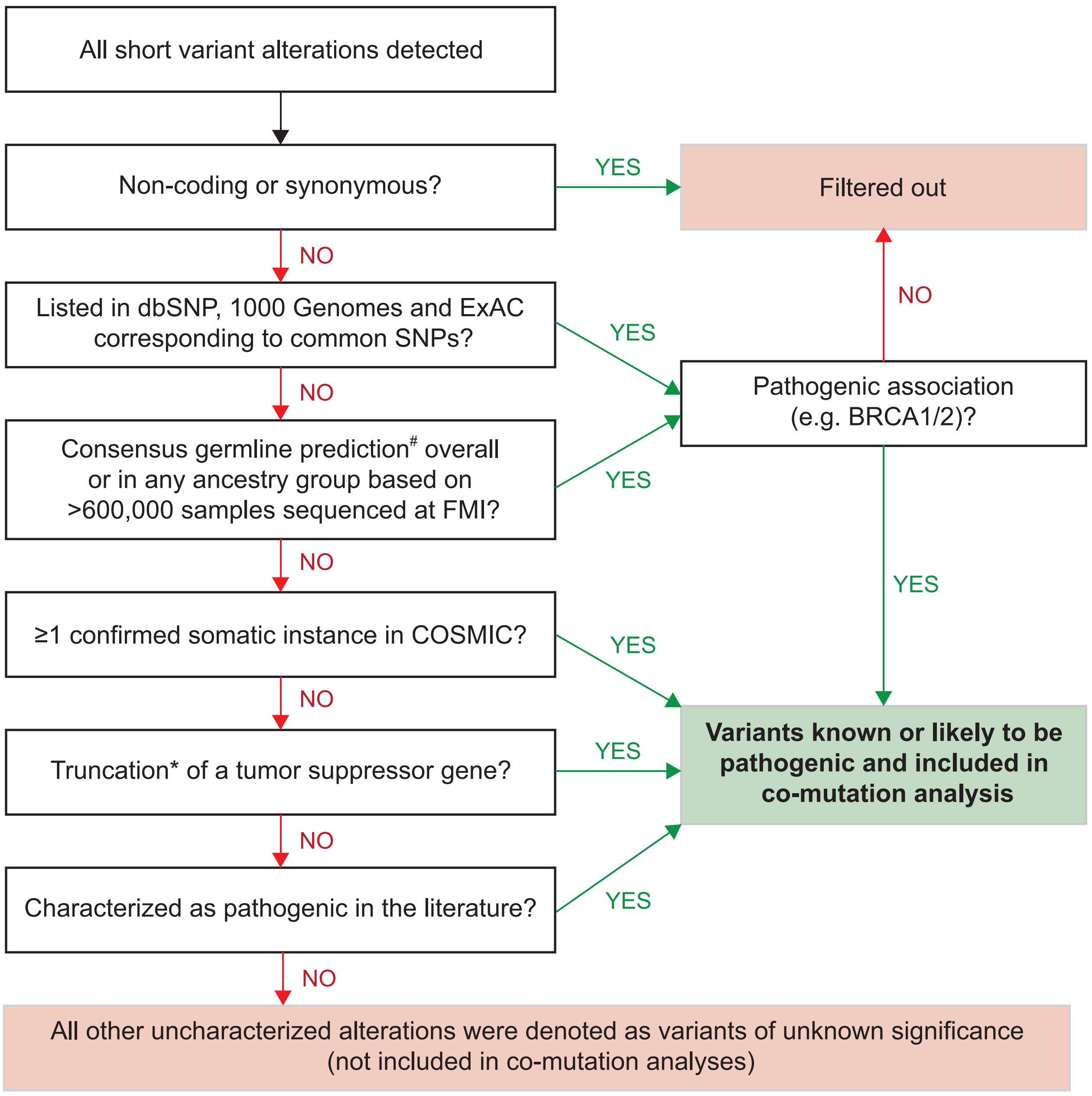

Short variant: single nucleotide variant or insertion or deletion. VUS: variant of unknown significance; FM: Foundation Medicine. *Including nonsense, frameshift, splice or deletion of a tumor suppressor predicted to result in loss of function. #Germline prediction performed using the SGZ algorithm described in Methods.
